# Analysis of global, regional, and national burden and attributable risk factors of acute lymphoblastic leukemia and acute myeloid leukemia from 1990 to 2021

**DOI:** 10.1371/journal.pone.0330479

**Published:** 2025-09-02

**Authors:** Haojie Ni, Yun Shi, Minyan Wang, Conghua Ji

**Affiliations:** School of Public Health, Zhejiang Chinese Medical University, Hangzhou, Zhejiang, China; Celal Bayar University: Manisa Celal Bayar Universitesi, TÜRKIYE

## Abstract

**Background:**

Acute Leukemia (AL) is a prevalent subtype of leukemia, mainly including acute lymphoblastic leukemia (ALL) and acute myeloid leukemia (AML). The disease burden of acute leukemia has significantly shifted in recent years. The aim of this study was to evaluate global trends in the burden of disease for ALL and AML from 1990 to 2021.

**Methods:**

Data on ALL and AML, encompassing incidence, mortality, disability-adjusted life-years (DALYs), and associated risk factors from 1990 to 2021, were extracted from the Global Burden of Disease (GBD) 2021 database. Estimated annual percentage changes (EAPC) were calculated to assess the changes in age-standardized incidence rate (ASIR), age-standardized mortality rate (ASMR), and age-standardized DALYs rate (ASDR). The associations between cancers burden and socio-demographic index (SDI) were also analyzed.

**Results:**

Compared with 1990, the global incidence of ALL and AML in 2021 is 5.69% and 82.25% higher, respectively. During the period from 1990 to 2021, ASMR in ALL showed a large decline, while AML remained stable. The ASDR of both showed a downward trend (EAPC = −2.11% and −0.84%). Regions and countries with higher SDI also have higher rates of acute leukemia. The burden of AML is mainly distributed in the elderly population, while the burden of ALL is heavier in children. Smoking, high BMI, and occupational exposure to benzene and formaldehyde are major risk factors for AML and ALL-related deaths.

**Conclusion:**

Acute leukemia remains one of the major global public health challenges, but there are different trends in different regions and countries. Acute myeloid leukemia has had a higher disease burden in recent years than acute lymphoblastic leukemia. Policy makers should develop targeted public health policies to further reduce the global burden of acute leukemia.

## 1. Introduction

Hematologic malignancies significantly contribute to the global cancer burden, with leukemia being prevalent among them. By 2022, leukemia is projected to rank as the 13^th^ most prevalent cancer globally and the 10^th^ leading cause of cancer-related mortality [1]. Leukemia manifests in two primary subtypes based on onset: acute and chronic. Acute Leukemia (AL) represents a malignant clonal disorder of hematopoietic stem cells typified by aberrant differentiation and uncontrolled proliferation [2]. Compared with chronic leukemia, AL has a short natural course and rapid onset, with infection, bleeding, anemia, and extramedullary tissue and organ infiltration as the main clinical manifestations. In 1976, The French-American-British (FAB) Cooperative Group distinctly classified AL into acute lymphoblastic leukemia (ALL) and acute myeloid leukemia (AML) [3].

AML is a bone marrow cancer characterized by uncontrolled proliferation of bone marrow stem cells, which can lead to infection, anemia, bleeding, and other symptoms [4]. AML predominantly affects the elderly, with approximately 80% of new cases reported in individuals aged 60 and above [5,6], boasting the highest fatality rate among various leukemia subtypes [7]. ALL is a malignant transformation and proliferation of lymphoblastic progenitor cells in bone marrow, blood, and extramedullary locations, and more than 80% of ALL occurs in children [8].

Different acute leukemia subtypes have different mortality, morbidity, prevalence, and disability-adjusted life-years (DALYs) [9]. Despite the emergence of novel therapeutic approaches in leukemia management, such as mutation-targeted inhibitors, pro-apoptotic agents, and immunotherapies, alongside significant advancements in bone marrow stem cell transplantation – including improved high-resolution HLA typing and matching, enhanced anti-infective strategies, and reduced-intensity conditioning protocols – these developments have significantly improved survival rates [10]. They effectively reduce graft-versus-host disease (GVHD) and infection-related mortality, collectively contributing to long-term remission. However, leukemia continues to impose a significant burden on patients [11,12]. Although several studies have reported the epidemiological characteristics of some subtypes of leukemia, the burden of leukemia has not been fully assessed [13,14].

The Global Burden of Disease (GBD) stands as the primary data repository for evaluating disease burdens, offering a comprehensive view of global, regional, and national health by analyzing health metrics across various causes over extended durations [15]. Previous studies utilizing GBD data have estimated the leukemia burden [16,17]. For example, analyses based on GBD 2019 indicated a declining trend in the global leukemia burden between 1990 and 2019, though levels remained relatively high overall [18]. However, these studies lacked a specific focus on acute leukemia subtypes, were confined to only selected populations and specific regions, and exhibited an absence of subtype-specific analyses using GBD 2021 data [19]; consequently, the research findings lack generalizability. Consequently, the present study leveraged the GBD 2021 database to systematically assess theincidence, mortality, DALYs, and estimated annual percentage changes (EAPC) of ALL and AML across global, national (204 countries and territories), and regional (21 regions) levels. This analysis delineates the current status and regional disparities in the burden of AL. Further stratification by sex, age group, and Socio-demographic Index (SDI) region was performed to identify key populations predominantly affected by distinct acute leukemia subtypes and to delineate temporal trends in disease burden. Attributable risk factors for mortality were also identified. The findings aim to inform policymakers and clinicians in formulating appropriate prevention and management strategies. In summary, comprehensive epidemiological assessment of global acute leukemia subtypes enhances understanding of disease distribution and trends, thereby facilitating improved quality of patient care, optimized resource allocation, and the development of targeted public health measures.

## 2. Methodology

This is a study based on public databases and does not involve participants, therefore it does not require ethics committee approval and is not applicable to patient informed consent.

### 2.1 Data sources

The data for this study came from the publicly available GBD 2021 database, which has been updated and expanded from GBD 2019 and provides the most recent global disease data. A total of 204 countries and territories are included in the database and are grouped into 21 regions based on epidemiological homogeneity and geographic proximity. Regions around the world are divided into five groups based on the SDI, a composite indicator based on per capita income, average educational attainment and total fertility. The GBD 2021 database systematically estimated incidence, prevalence, mortality, years of life lost (YLLs), years of life lost (YLDs), and DALYs for 369 diseases and injuries from 1990 to 2021. Input data were extracted from censuses, household surveys, civil registration and vital statistics, disease registries, health service use, air pollution monitors, satellite imaging, disease notifications, and other sources [20]. We used the GBD 2021 database to extract information on the number of deaths, DALYs, and corresponding age-standardized rates (ASR) attributable to acute leukemia from 1990 to 2021. Data were analyzed by age, sex, location and year to get a complete picture of the disease burden caused by acute leukemia.

### 2.2 Acute leukemia definition

All acute leukemias were defined based on the International Classification of Diseases (ICD) diagnostic criteria. The associated ICD-9 and ICD-10 codes of ALL and AML for incidence and mortality data were as follows: ALL (C91.0-C91.02, C91.2-C91.32, C91.6-C91.62, 204.0–204.02, C91.0, C91.2-C91.3, C91.6), AML (C92.0-C92.02, C92.3-C92.62, C93.0-C93.02, C94.0-C94.02, C94.2-C94.22, C94.4-C94.5, 205.0–205.02, 205.2–205.32, 206.0–206.02, 207.0–207.02, 207.2–207.82, C92.0, C92.3-C92.6, C93.0, C94.0, C94.2, 205.0, 205.2–205.3, 206.0, 207.0, 207.2–207.8). The incidence, mortality, and DALYs estimation for acute leukemias were described in the GBD 2021 study.

### 2.3 Attributable risk factors

GBD 2021 estimated the attributable disease burden of 88 risk factors and combinations of risk factors at the global, regional, and national levels. Attributable DALY is the reduction in current disease DALY that would have been possible if exposure to the risk factor at the population level had shifted to an alternative or counterfactual distribution of risk. Similarly, attributable deaths represent the reduction in current deaths that would have been possible if the population had been exposed to a counterfactual level of the theoretical minimum risk exposure. Attributable DALY and attributable deaths were estimated by multiplying the total DALY or death count of a specific outcome by the population attributable fraction. The population attributable fraction represents the proportion of outcomes that would decrease in a given population and time if exposed to a counterfactual level of the theoretical minimum risk exposure level. It provides an understanding of how much of the disease burden or deaths can be attributed to a specific risk factor. Other published literature also provides definitions of risk factors and specific estimation methods [21].

Attributable risk factors in the analysis included Smoking, High BMI, Occupational exposure to benzene, and Occupational exposure to formaldehyde. Percentage of AML and ALL-related deaths and DALYs are available in the GBD outcome tool.

### 2.4 Statistical analysis

ASR is a statistical measure used to compare disease incidence or mortality across different populations or regions. By weighting the morbidity or mortality of each population or region according to a certain standard age structure, ASR eliminates the influence of differences in age structure, making the comparison results more comparable. The ASR (per 100,000 populations) was calculated using the following formula:


$$ASR=\frac{\sum_{i=1}^{A}a_{i}w_{i}}{\sum_{i=1}^{A}w_{i}}\times \text{100,000}$$

In the formula, α_i_ denotes the age‐specific rates in the i^th^ age group, w_i_ denotes the number of persons (or the weight) in the corresponding i^th^ age subgroup of the selected reference standard population, and A denotes the number of age groups.

To describe the trend of ASR within a specific time interval, EAPC were utilized. A regression line was employed to estimate the natural logarithm of the rates, given by the equation y = α + βx + ε, where y = ln (ASR) and x = calendar year. The EAPC was calculated as 100 × [exp(β) – 1], accompanied by a 95% confidence interval (CI) derived from the linear regression model. An increasing trend was observed when the EAPC value and its 95% CI were > 0, while a decreasing trend was observed when the EAPC value and its 95% CI were < 0. All statistical analyses and figures were performed using R software (version 3.6.3). Supplementary Figure 1 shows the analysis process of the entire research.

## 3. Results

### 3.1 Global incidence, mortality and DALYs

In 2021, the global incidence of ALL was 103.73 × 10^3^, and that of AML was 144.65 × 10^3^. In comparison to 1990, there was a substantial increase in the incidence of AML (82.25%), significantly surpassing that of ALL (5.69%) (Table 1). From 1990 to 2021, the age-standardized incidence rate (ASIR) of AML was higher than that of ALL, and the gap between the two was also increasing, with significant fluctuations beginning in 2019. The all-ages incidence rate and all-ages mortality rate of AML showed an upward trend during 1990–2021, while its ASIR and age-standardized mortality (ASMR) showed a relatively stable trend globally (S2 Fig).

**Table 1:** Global incidence, mortality and DALYs of acute leukemia from 1990 to 2021.

	Acute lymphoblastic leukemia			Acute myeloid leukemia		
	Both	Male	Female	Both	Male	Female
1990*						
Incident cases (×10^3^)	98.15(77.90,123.51)	53.31(35.69,72.04)	44.84(31.9,59.83)	79.37(62.81,99.43)	41.41(30.81,54.09)	37.96(30.06,46.59)
Deaths (×10^3^)	82.77(64.01,105.55)	45.13(29.19,62.80)	37.64(25.83,50.97)	74.92(58.73,94.71)	39.17(28.89,51.12)	35.74(28.15,44.48)
DALYs (×10^4^)	545.51(414.97,713.52)	299.31(178.83,423.86)	246.2(164.83,341.77)	334.29(239.42,470.91)	177.82(116.50,265.83)	156.47(106.90,206.77)
ASIR^*^ (1/10^5^)	1.79(1.42,2.24)	1.94(1.32,2.62)	1.65(1.16,2.18)	1.77(1.43,2.16)	1.98(1.55,2.44)	1.62(1.32,1.97)
ASMR^*^ (1/10^5^)	1.55(1.21,1.96)	1.70(1.14,2.35)	1.41(0.97,1.90)	1.69(1.36,2.08)	1.91(1.48,2.35)	1.53(1.24,1.88)
ASDR^*^ (1/10^5^)	94.89(72.48,123.29)	102.56(63.09,143.80)	87.23(59.11,120.68)	65.55(48.34,88.77)	70.84(49.19,99.70)	60.98(43.3,78.93)
2021*						
Incident cases (×10^3^)	103.73(71.61,122.52)	60.91(38.15,78.20)	42.82(26.56,50.24)	144.65(126.24,164.85)	77.25(62.28,92.90)	67.39(57.63,80.21)
Deaths (×10^3^)	71.22(49.17,83.17)	41.78(25.89,54.52)	29.44(17.87,34.95)	130.19(113.63,149.39)	70.48(56.52,85.81)	59.71(50.83,71.70)
DALYs (×10^4^)	371.98(258.75,435.32)	223.12(140.98,283.61)	148.86(92.23,174.31)	413.51(344.65,489.52)	221.23(161.48,280.09)	192.28(157.41,243.00)
ASIR^*^ (1/10^5^)	1.37(0.95,1.64)	1.61(1.01,2.06)	1.14(0.71,1.35)	1.73(1.51,1.98)	1.99(1.62,2.38)	1.53(1.30,1.83)
ASMR^*^ (1/10^5^)	0.90(0.62,1.05)	1.07(0.67,1.40)	0.73(0.45,0.86)	1.57(1.37,1.80)	1.84(1.48,2.22)	1.35(1.15,1.64)
ASDR^*^ (1/10^5^)	48.86(34.15,57.46)	58.06(36.58,73.54)	39.44(24.65,46.42)	50.79(42.16,60.37)	55.82(40.70,71.02)	46.55(37.99,59.22)
**1990-2021**						
ASIR EAPC (95% *CI*)	−0.64% (−0.74, −0.54)	−0.40% (−0.49, −0.30)	−0.98% (−1.08, −0.88)	−0.03% (−0.12, 0.06)	0.09% (0.00, 0.17)	−0.16% (−0.25, −0.07)
ASMR EAPC (95% *CI*)	−1.73% (−1.76, −1.70)	−1.44% (−1.47, −1.41)	−2.12% (−2.15, −2.09)	−0.21% (−0.29, −0.14)	−0.06% (−0.14, 0.02)	−0.39% (−0.47, −0.31)
ASDR EAPC (95% *CI*)	−2.11% (−2.15, −2.06)	−1.79% (−1.84, −1.74)	−2.54% (−2.58, −2.49)	−0.84% (−0.89, −0.78)	−0.77% (−0.82, −0.72)	−0.9% (−0.97, −0.84)

All data reported as number or rate (95% UI); ^*^Annual age-standardized rates (per 100,000 population)

AML will cause 130.19 × 10^3^ cases of death globally in 2021, 1.83 times more than ALL (Table 1). From 1990 to 2021, ASMR for ALL showed a large decline, while ASMR for AML remained stable (S2 Fig). In the past 30 years, only male patients with AML had stable changes in ASMR EAPC (EAPC = −0.06%; 95% CI: −0.14, 0.02), while the rest showed a downward trend.

In 2021, the DALYs of AML were 413.51 × 10^4^, slightly higher than that of ALL (Table 1). The age-standardized DALYs rate (ASDR) of both groups showed a downward trend, and the ASDR of ALL showed a greater decline, from being higher than that of AML in 1990, to basically flat in 2018, and then lower than that of AML (S2 Fig). The EAPC decline in ASDR was most pronounced in female patients with ALL (EAPC = −2.54%; 95% CI: −2.58, −2.49, Table 1).

### 3.2 Regional incidence, mortality and DALYs

**Fig 1:**
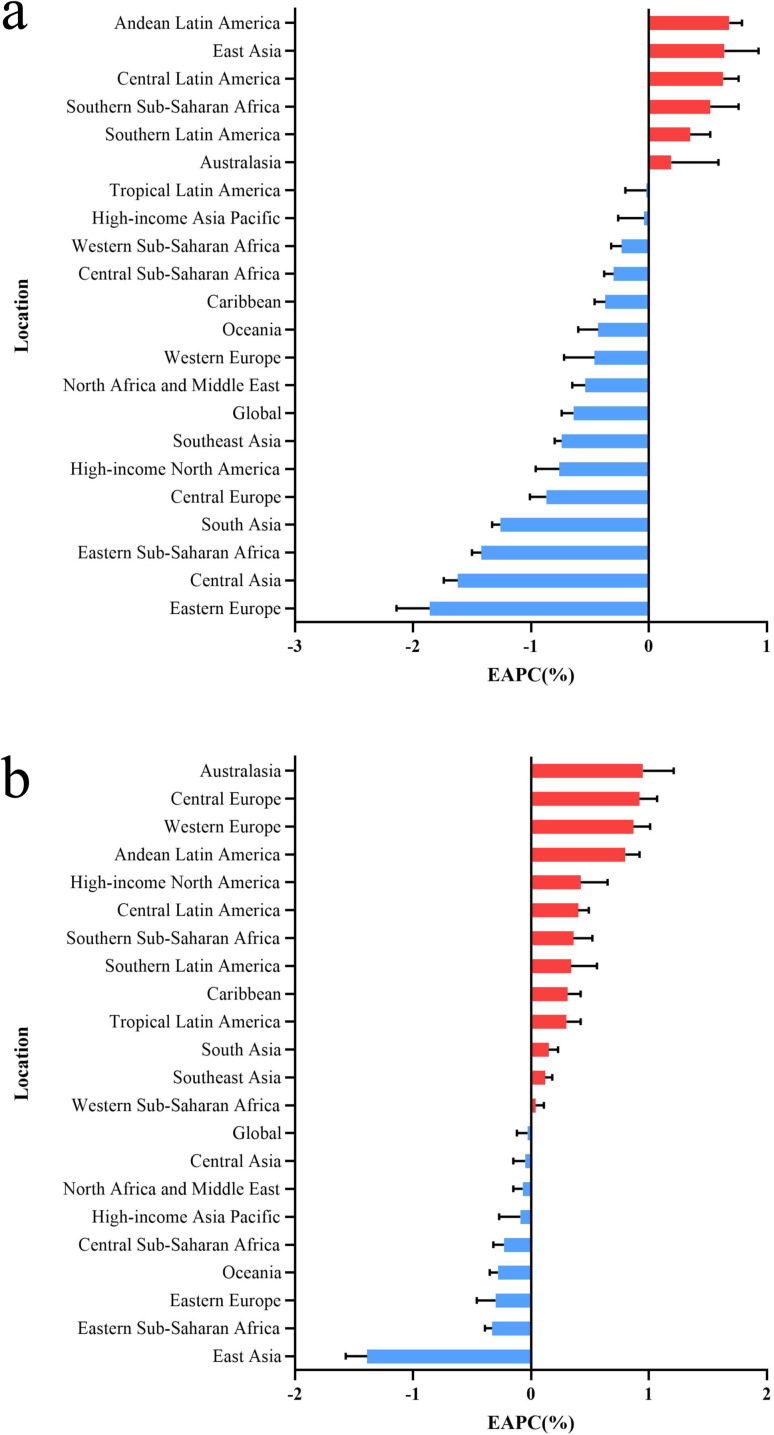
EAPC of ASIR for acute leukemia in global and 21 regions. (a) ALL (b) AML.

In 2021, The regions with the highest number of ALL cases were East Asia (39.30 × 10^3^), South Asia (11.45 × 10^3^) and Southeast Asia (8.35 × 10^3^) (S1 Table). These three regions also have a high incidence of AML, but the largest number of AML cases is still in Western Europe (24.64 × 10^3^). In addition, ASIR of ALL and AML showed a significant upward trend in both Andean Latin America and Central Latin America. In Eastern Europe and Eastern Sub-Saharan Africa, there is a more obvious downward trend (Fig 1). The ASIR of ALL patients in East Asia showed a great upward trend, while the ASIR of AML in this region showed a great downward trend.

In 2021, East Asia and South Asia had the highest number of deaths from ALL and AML in all 21 regions. The highest ASMR for ALL and AML was 1.83 per 100,000 persons in Andean Latin America and 2.85 per 100,000 persons in High-income North America (S2 Table). The trend of ASMR in all regions of AML is varied, while ALL is only on the rise in Southern Sub-Saharan Africa and Central Latin America (S3 Fig).

In 2021, the three regions with the highest DALY for ALL and AML were East Asia, South Asia and Southeast Asia. However, the region with the highest DALY was East Asia (951.38 × 10^3^), while the region with AML was South Asia (619.34 × 10^3^). The highest ASDR for ALL and AML were 101.43 per 100,000 persons in Central Latin America and 79.86 per 100,000 persons in Oceania (S3 Table). From 1990 to 2021, ASDR in acute leukemia is generally declining in all regions, with only Sub-Saharan Africa maintaining an upward trend in ASDR (S4 Fig).

### 3.3 National incidence, mortality and DALYs

In 2021, China (38,570.94) and the USA (21,533.02) had the highest number of cases of ALL and AML, respectively. Similarly, among the 204 countries and regions, China (20,612.91) and the USA (16,648.46) had the highest number of deaths from ALL and AML, respectively. China’s ALL (924,422.19) and AML (548,555.39) also resulted in the highest DALYs in the world, 2.33 times and 1.25 times those of India, which ranked second, respectively (S4 Table).

Trends in ASIR, ASMR, and ASDR vary widely across the 204 countries and territories. Ghana (EAPC = −2.99%; 95% CI: −3.49, −2.49) and Mauritius (EAPC = 5.12%; 95% CI:1.91, 8.43) have the largest ASIR changes in ALL and AML, respectively (S5 Table). Belarus (EAPC = −4.13%; 95% CI: −4.67, −3.58) and Mauritius (EAPC = 5.15%; 95% CI:1.91, 8.50) had the highest amplitude of ASMR changes in ALL and AML, respectively (S6 Table). Belarus (EAPC = −4.81%; 95% CI: −5.35, −4.26) and Mauritius (EAPC = 3.49%; 95% CI:e0.82, 6.22) had the highest amplitude of ASDR change in ALL and AML, respectively (S7 Table). AML ASIR and ASMR in Mauritius have the highest growth rate in the world from 1990 to 2021.

### 3.4 Burden of acute leukemia by SDI

From 1990 to 2021, ALL had the highest number of incident cases, deaths, and DALYs in Middle SDI region, while AML has the highest number of incident cases and deaths in High SDI region. Both ALL and AML have lower ASR in areas with Low SDI region. The ASR of all SDI regions of ALL showed a relatively obvious downward trend, while that of AML showed a relatively stable trend (S5 and S6 Figs).

**Fig 2:**
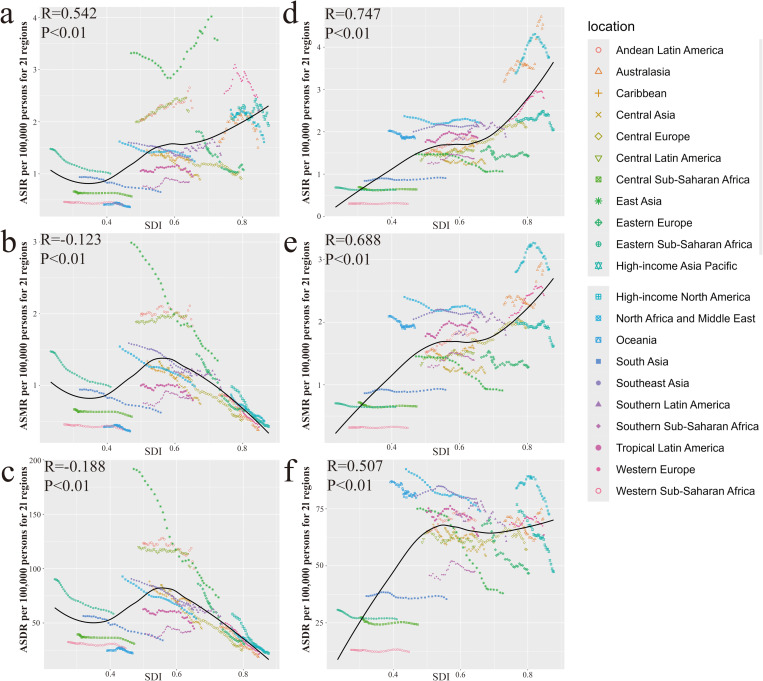
ASR of acute leukemia for 21 regions by SDI. (a) ALL ASIR (b) ALL ASMR (c) ALL ASDR (d) AML ASIR (e) AML ASMR (f) AML ASDR.

**Fig 3:**
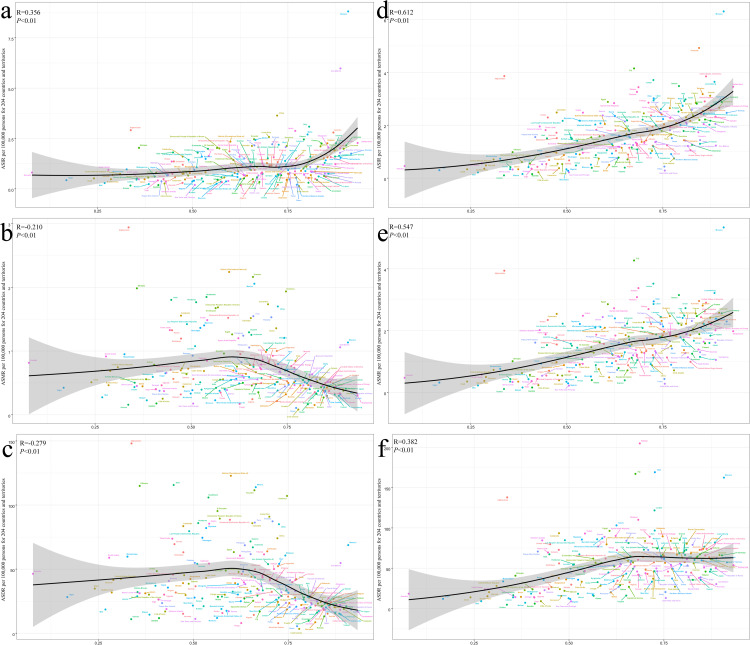
ASR of acute leukemia for 204 countries and territories by SDI. (a) ALL ASIR (b) ALL ASMR (c) ALL ASDR (d) AML ASIR (e) AML ASMR (f) AML ASDR.

The ASIR of the high-level SDI region ALL is also relatively high. However, ASMR and ASDR of ALL showed an overall downward trend as SDI increased. The ASIR, ASMR and ASDR of AML in the High-level SDI region were all higher than those in the Low-level SDI region. At regional and national levels, ALL is associated with ASIR, ASMR, ASDR, and SDI in AML. In all of the 204 countries and territories, ASR in Monaco for acute leukemia is well above the level expected in the region in 2021(Figs 2 and 3).

### 3.5 Burden of acute leukemia by age and sex

**Fig 4:**
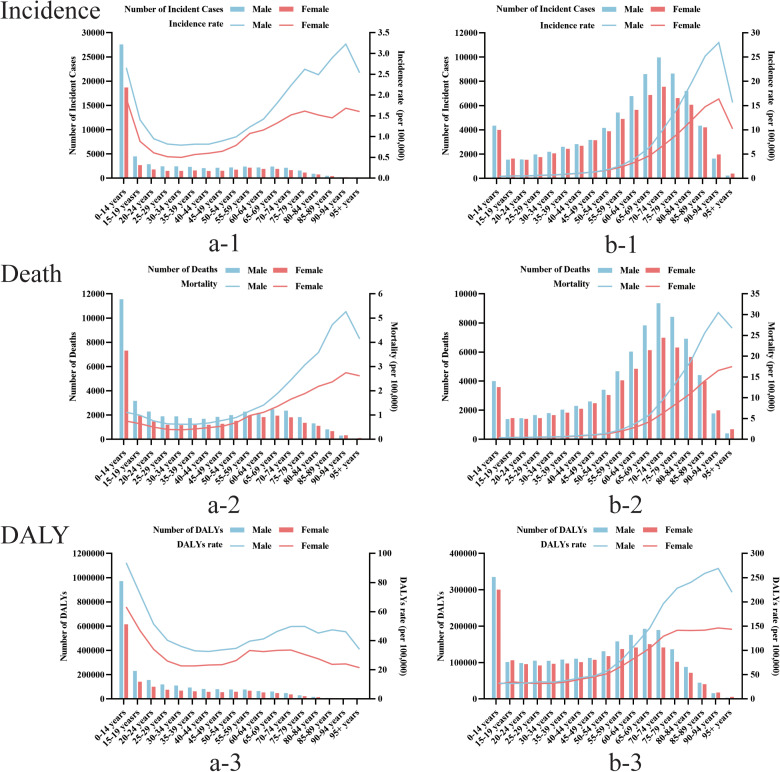
Based on the gender group of a comparison of the age distribution of global acute leukemia. (a) ALL (b) AML.

In 2021, the 0–14 years age group had the most ALL incident cases, deaths and DALYs, far more than any other age group. incident cases and deaths from AML increase with age, peaking at 70–74 years of age and then declining. The incidence rate, mortality rate and DALYs rate of AML also increase with age, and show a big turning point at the age of 90–94, and then start to show a downward trend. Notably, there are also gender differences in the burden of AL disease among different age groups globally, with an overall situation where men have a higher burden than women (Fig 4).

**Fig 5:**
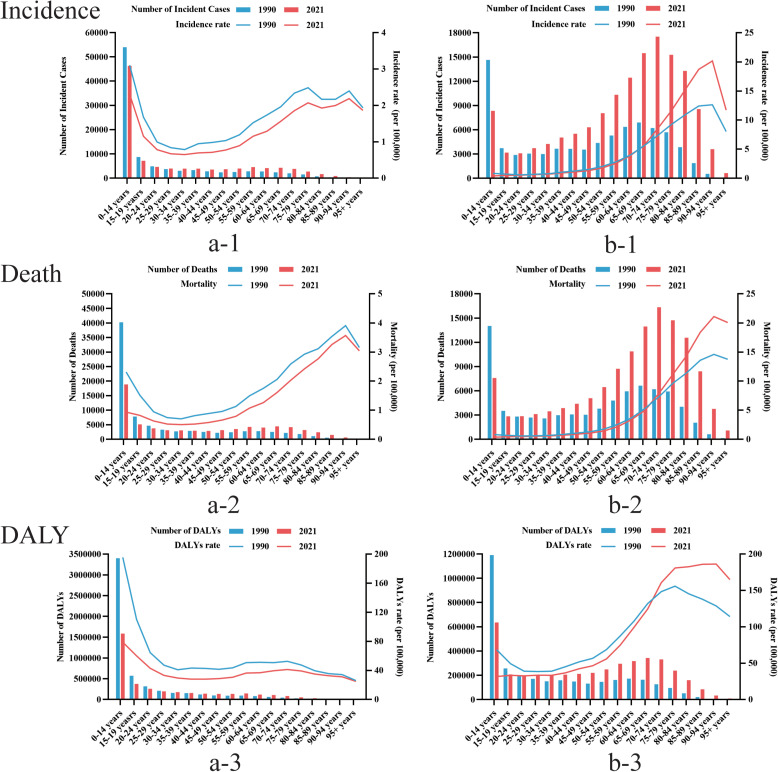
Based on the time group of a comparison of the age distribution of global acute leukemia. (a) ALL (b) AML.

Compared with 1990, the global burden rate of ALL diseases generally decreased in 2021. Among them, the disease burden of ALL among the younger age group showed a greater absolute decline, especially in terms of death and DALY. For AML, although the disease burden has decreased in the younger age group, for the middle-aged and elderly group, especially patients over 70 years old, the disease burden has increased (Fig 5).

### 3.6 Attributable risk factors

Smoking, High BMI, Occupational exposure to benzene and Occupational exposure to formaldehyde were the top four risk factors for acute leukemia. smoking and High BMI were the main attributed risk factors. The proportion of acute leukemia deaths and DALYs due to all risk factors increased in 2021 compared to 1990. In 2021, 5.68% of ALL deaths and 2.79% of DALYs could be attributed to smoking, and 11.47% of AML deaths and 7.47% of DALYs could be attributed to smoking (S8 Table).

**Fig 6:**
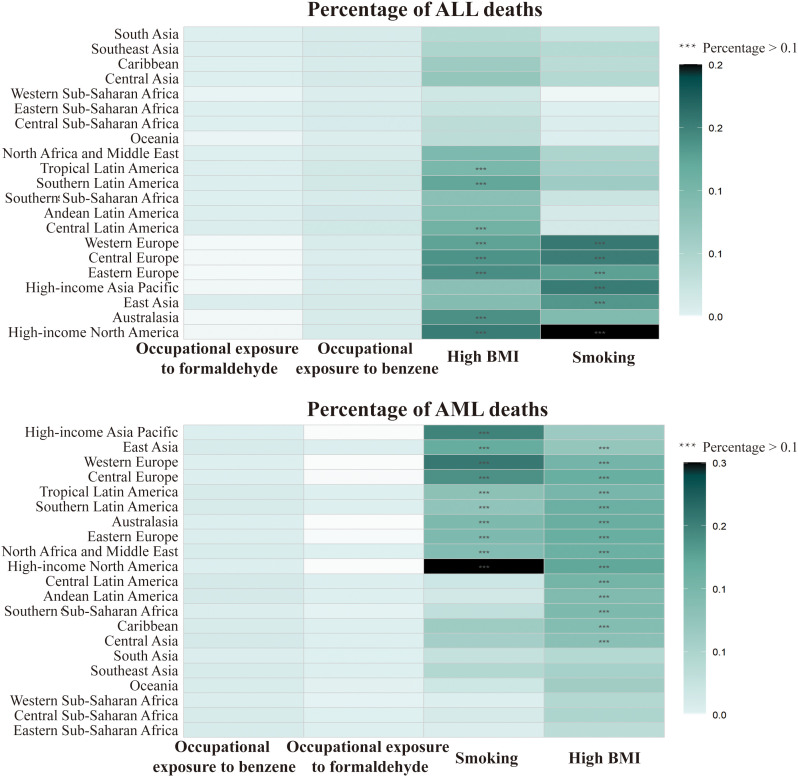
Leading 4 Level 3 causes of global incident acute lymphoblastic leukemia and acute myeloid leukemia deaths in 2021.

In 2021, a large proportion of ALL and AML-related deaths could be attributed to Smoking and High BMI (percentage>0.1) in all European regions, while a lower proportion was observed in all Sub-Saharan Africa regions. ALL Latin America regions have a larger proportion of AML-related deaths (percentage>0.1) due to Smoking and High BMI, compared to a smaller proportion for ALL (Fig 6).

## 4. Discussion

This study reports morbidity, mortality, and DALY data for acute leukemia from the GBD 2021 database. Moreover, we examined epidemiological trends in AL by calculating the Estimated Annual Percentage Change values over the past 32 years. Overall, the total number of cases and the total number of deaths increased globally for different subtypes of AL, however, the ASR for different subtypes of AL showed an overall downward trend, likely due to effective diagnostic and treatment strategies, which is similar to previous reports [22]. In comparison to ALL, AML exhibits a wider range of variability. The AML all-ages incidence rate and all-ages mortality rate showed an increasing trend during 1990–2021. However, its age-standardized incidence rate and age-standardized mortality rate are relatively stable. Mainly due to the high incidence of AML in elderly patients, the incidence gradually increases with age [23]. The aging of the population has brought about changes in the age structure, with the proportion of the elderly increasing and the proportion of the young decreasing. Thus, despite the increase in overall incidence, the age-standardized incidence may remain unchanged. The high incidence in older age groups may offset changes in other age groups. Given this trend, integrating early screening for AML into routine health management systems for individuals aged 65 and older is recommended. As the global population ages, it is necessary to pay attention to the rapid growth of AML. Although the trends in ASIR and ASMR were different in both diseases, ASDR showed a downward trend in both diseases, possibly due to breakthroughs in treatment methods (such as the application of targeted drugs and immunotherapy), the optimization of supportive treatment, and new chemotherapy regimens [24–26]. Enhancing global accessibility of novel therapeutic regimens should be prioritized to narrow treatment disparities in low- and middle-income regions. This trend aligns with ASDR trajectories observed in other GBD studies (1990–2019), reflecting the persistent global decline in AL burden [16].

In addition to age, sex is also an important factor affecting AL epidemiology. Both AML and ALL patients always have a higher incidence in men than in women. The incidence of AL in males is generally higher than that in females, with an overall male-to-female ratio of 1.4 [27]. This sex difference may be due to the fact that men are more likely to develop cancer, especially hematologic malignancies [28]. In this study, we found that occupational exposure to smoking, benzene, or formaldehyde were major risk factors for AL-related death, which may also contribute to the higher disease burden in men. Smoking has been identified as a major risk factor for leukemia, causing a prevalence of 25.0% in men, which is five times higher than that of women (5.4%) [29]. In the burden of leukemia disease caused by smoking, significant gender differences can be observed. It is urgently necessary to implement a comprehensive smoking ban policy in the workplace in male-dominated industries (such as manufacturing and transportation), and incorporate blood routine monitoring into the mandatory occupational health examination items. This is also consistent with the research results of GBD 2019 [18]. We also investigated potential risk factors for AL-related deaths, and in addition to the risk factors mentioned above, high BMI was the second highest risk factor after smoking. Previous studies have also confirmed that being overweight or obese is a poor prognostic predictor for some AML subtypes and a marker of early death in ALL patients [30,31]. Clinical guidelines should incorporate standardized weight management protocols for leukemia patients, with metabolic health management integrated throughout the cancer care continuum. Beyond risk factors captured in the GBD database, genetic risk factors constitute significant determinants of AL pathogenesis. Substantial evidence indicates that specific genetic variants act as independent drivers of hematopoietic stem cell malignant transformation, directly initiating ALL or AML. For instance, Down syndrome (trisomy 21) constitutes an established independent genetic risk factor for pediatric ALL, substantially elevating disease susceptibility [32]. Similarly, inherited disorders such as Fanconi anemia and neurofibromatosis type 1 (NF1), mediated by specific pathogenic germline mutations in genes including those within the BRCA pathway or NF1, independently and substantially elevate the risk of developing specific AML or ALL subtypes [33,34].

There are also significant differences in incidence, mortality, and DALY among different regions and countries. Central Latin America exhibits the highest ASDR for ALL among all regions, a pattern likely attributable to widespread agricultural pesticide exposure in intensive farming practices. Elevated acute leukemia risks are associated with occupational pesticide exposure [35], and within this region, substantial glyphosate application in soybean and maize cultivation may significantly contribute to the observed disease burden disparities. Accelerating agricultural modernization through policy-driven reduction of carcinogenic agrochemical use is therefore imperative. Asia has a high number of AML cases. The prevalence of hepatitis C in the region may partly explain the high incidence of AML [36]. The trend of ASMR and ASDR in AML with different SDI levels was relatively stable during the study period, which may be due to improved early diagnosis and the implementation of newer chemotherapy and treatment regimens [24]. ALL has a higher ASIR and a lower ASMR and ASDR in the High SDI area. Adequate medical resources and health care systems in the High SDI area play a certain role, while for some low-income areas, the basic intervention measures for ALL are different, which also leads to the lower survival rate in the region. Notably, ASIR, ASMR, and ASDR remained low in low-middle SDI and low SDI regions for both diseases. This may be due to limited data on local cancer registries, or lower levels of diagnosis. The international community needs to give priority to supporting the reconstruction of cancer diagnosis infrastructure in low-resource areas. In some countries, under-diagnosis and under-registration of cancer patients may also interfere with the AL disease burden. Of course, in parts of the world, such as Southern Sub-Saharan Africa, we have also observed more stable changes in the burden of disease, which may also be the result of improved local health infrastructure, international cooperation, and health assistance [37]. In Brazil, the primary health care system has also been instrumental in reducing the burden of acute leukemia [38]. In China, the mortality rate of AL and DALYs, especially DALYs, showed a significant downward trend, which was greatly influenced by the development of social economy and the improvement of health resources. China’s experience demonstrates that integrating healthcare systems with social development policies generates synergistic multiplier effects for cancer control, an approach meriting global consideration and adaptation. The policy-making of the healthcare system must also give priority to fair access to healthcare, improvement of infrastructure and training of healthcare personnel.

As with all GBD-related studies, a major limitation of this study is the inequity of medical diagnostic systems in different countries and regions, which can lead to misestimates of morbidity and mortality, resulting in overdiagnosis and missed diagnosis [39]. The diagnostic criteria and classification of hematological malignancies also vary from country to country and over time. Secondly, Coding biases, particularly from ICD transitions (e.g., ICD-9 to ICD-10 during 1990–2021), may compromise case identification consistency. These inconsistencies stem from divergent diagnostic criteria and coding rules across ICD versions, combined with asynchronous implementation timelines globally. Consequently, this heterogeneity could confound interpretations of epidemiological trends (e.g., incidence/mortality rates) and hinder valid cross-regional comparisons. Finally, our analysis was constrained to the risk factors included in GBD 2021, preventing a comprehensive exploration of all potential disease causes.

## 5. Conclusion

In summary, acute leukemia remains one of the major global public health challenges. This study found that from 1990 to 2021, ASIR, ASMR, and ASDR in acute leukemias are all decreasing globally. Men are at a higher risk. ALL is most common in children, while AML is most common in the elderly. The ASIR and SDI values for AL are positively correlated, which also means that the age-standardized incidence in developed regions is significantly higher than that in developing regions. Smoking, high body mass index, and occupational exposure to benzene and formaldehyde are ALL major risk factors for AML and ALL-related deaths, with varying risk levels in different regions. In addition, it is worth noting that AML has a higher disease burden globally than ALL. Therefore, policymakers should formulate targeted public health policies to further reduce the global burden of acute leukemia.

## Supporting information

S1 Fig Flow chart of the analysis. (TIF)

S2 Fig Global ASR and all-ages rate of acute leukemia from 1990 to 2021. (a) ASIR. (b) ASMR. (c) ASDR. (d) all-ages incidence rate (e) all-ages mortality rate (f) all-ages DALYs rate.
(TIF)

S3 Fig EAPC of ASMR for acute leukemia in global and 21 regions. (a) ALL (b) AML.
(TIF)

S4 Fig EAPC of ASDR for acute leukemia in global and 21 regions. (a) ALL (b) AML.
(TIF)

S5 Fig Burden of acute lymphoblastic leukemia by SDI quintile from 1990 to 2021. (TIF)

S6 Fig Burden of acute myeloid leukemia by SDI quintile from 1990 to 2021. (TIF)

S1 Table Regional incident cases and age-standardized incidence rate of acute leukemia in 2021. (DOCX)

S2 Table Regional deaths and age – standardized mortality rate of acute leukemia in 2021. (DOCX)

S3 Table Regional DALYs and age – standardized DALYs rate of acute leukemia in 2021. (DOCX)

S4 Table Incidence, mortality and DALYs of acute leukemia among the top three and bottom three countries in 2021. (DOCX)

S5 Table EAPC of ASIR for acute leukemia in 204 countries and territories from 1990 to 2021. (DOCX)

S6 Table EAPC of ASMR for acute leukemia in 204 countries and territories from 1990 to 2021. (DOCX)

S7 Table EAPC of ASDR for acute leukemia in 204 countries and territories from 1990 to 2021. (DOCX)

S8 Table Percentage of acute leukemia deaths and DALYs attributable to risk factors in 1990 and 2021. (DOCX)

## Abbreviations

AL: Acute leukemia
ALL: Acute lymphoblastic leukemia
AML: Acute myeloid leukemia;
ASDR: Age-standardized DALYs rate;
ASIR: Age-standardized incidence rate ;
ASMR: Age-standardized mortality rate;
ASR: Age-standardized rates;
BMI: Body mass index;
CI: Confidence interval;
DALYs: Disability-adjusted life-years;
EAPC: Estimated annual percentage changes;
GBD: Global Burden of Disease;
ICD: International Classification of Diseases;
SDI: Socio-demographic index;
UI: Uncertainty interval.
