# Supplementary figures and images for: Analysis of global, regional, and national burden and attributable risk factors of acute lymphoblastic leukemia and acute myeloid leukemia from 1990 to 2021
Source: PLoS One. 2025 Sep 2;20(9):e0330479. doi: 10.1371/journal.pone.0330479 (PMC12404455; doi:10.1371/journal.pone.0330479)

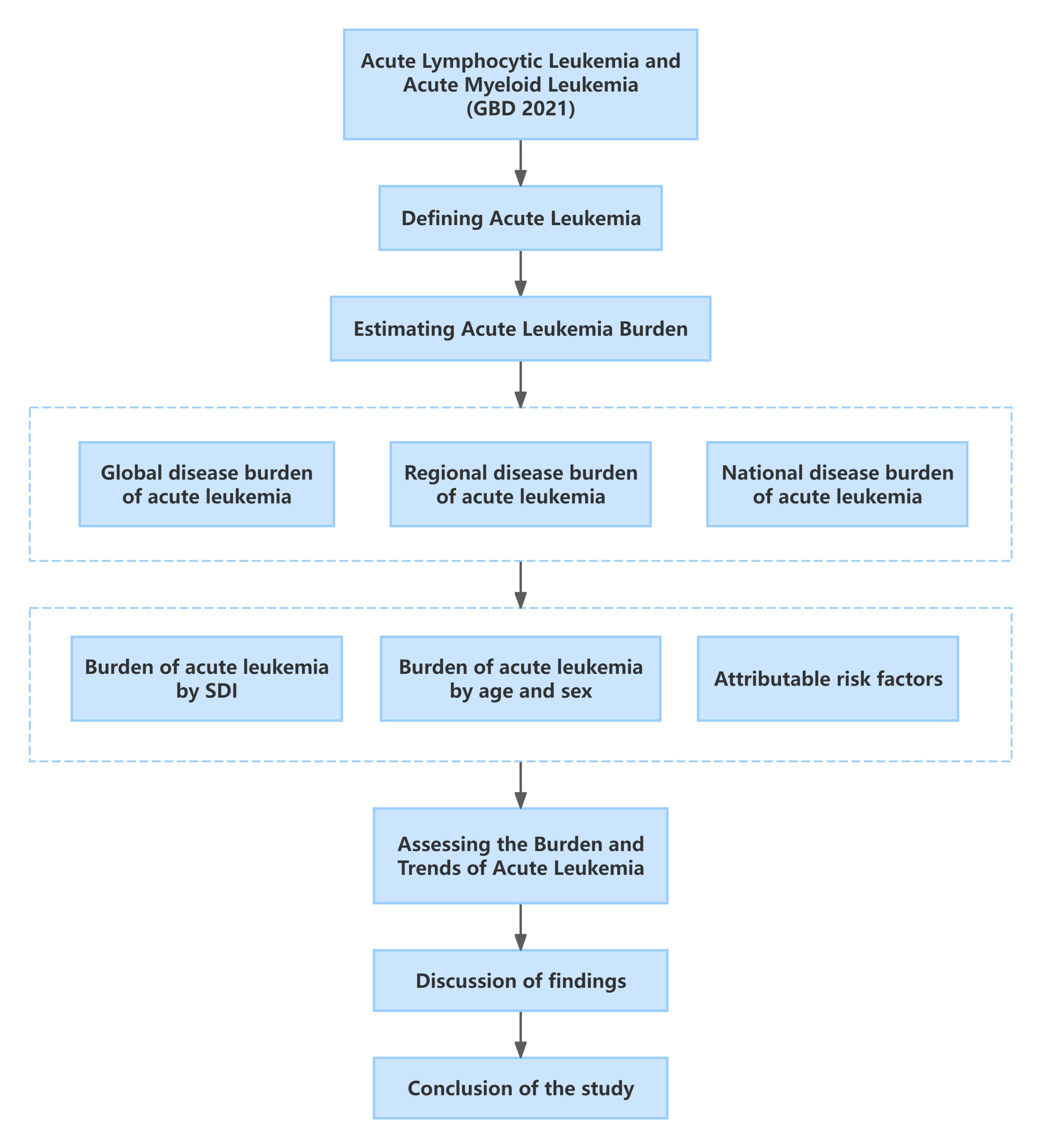

Supplement: S1 Fig — (TIF) [file pone.0330479.s001.tif]

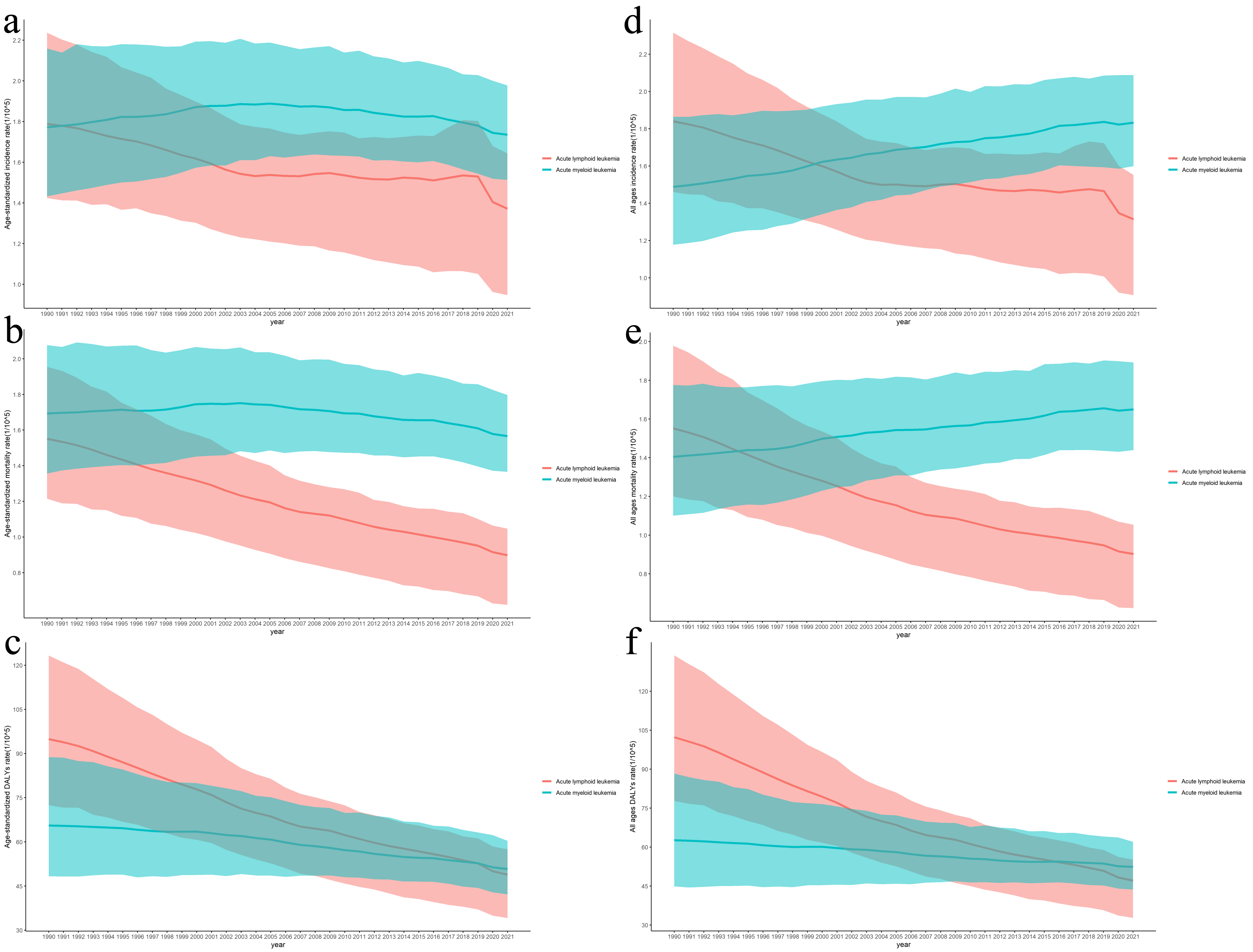

Supplement: S2 Fig — (a) ASIR. (b) ASMR. (c) ASDR. (d) all-ages incidence rate (e) all-ages mortality rate (f) all-ages DALYs rate. (TIF) [file pone.0330479.s002.tif]

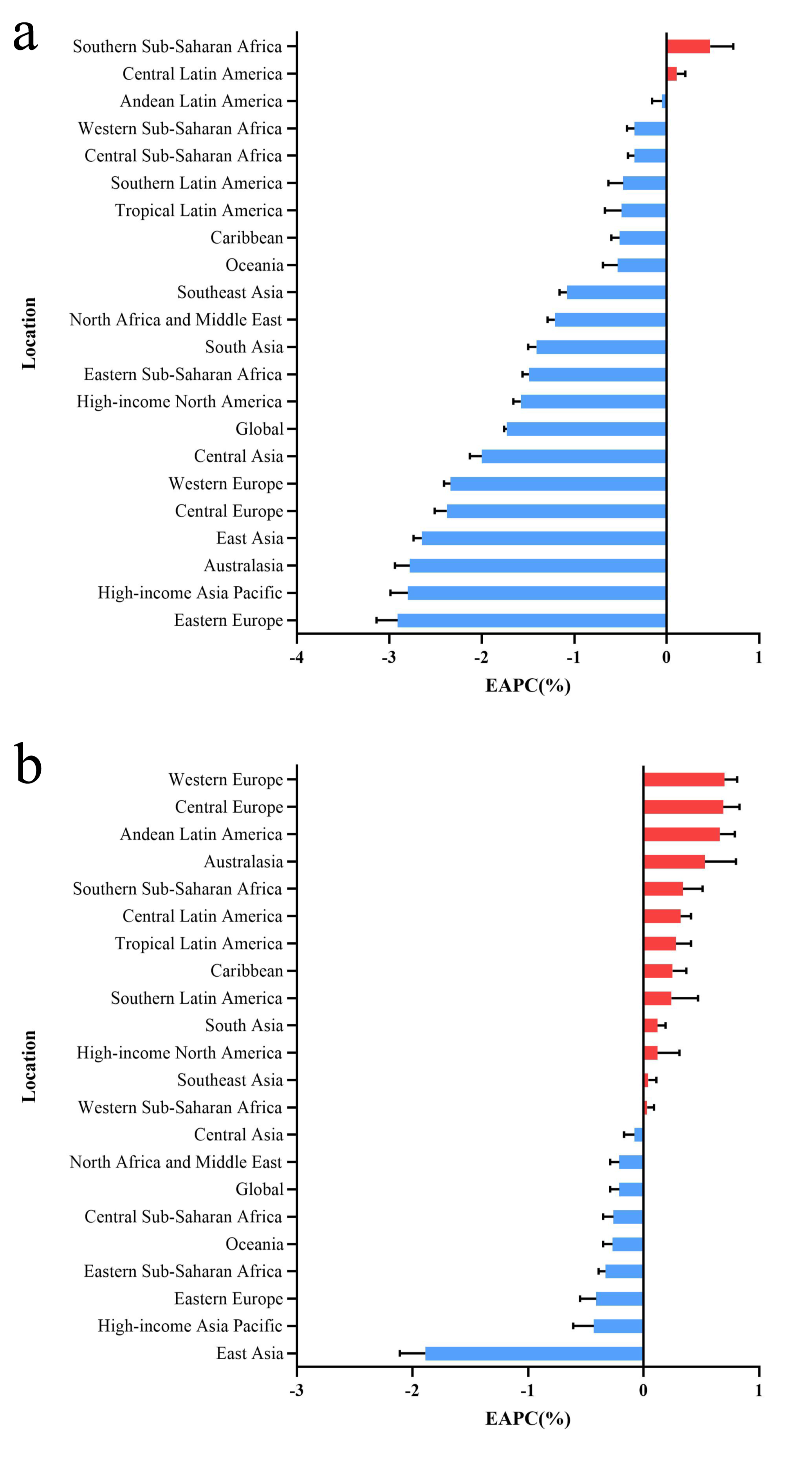

Supplement: S3 Fig — (a) ALL (b) AML. (TIF) [file pone.0330479.s003.tif]

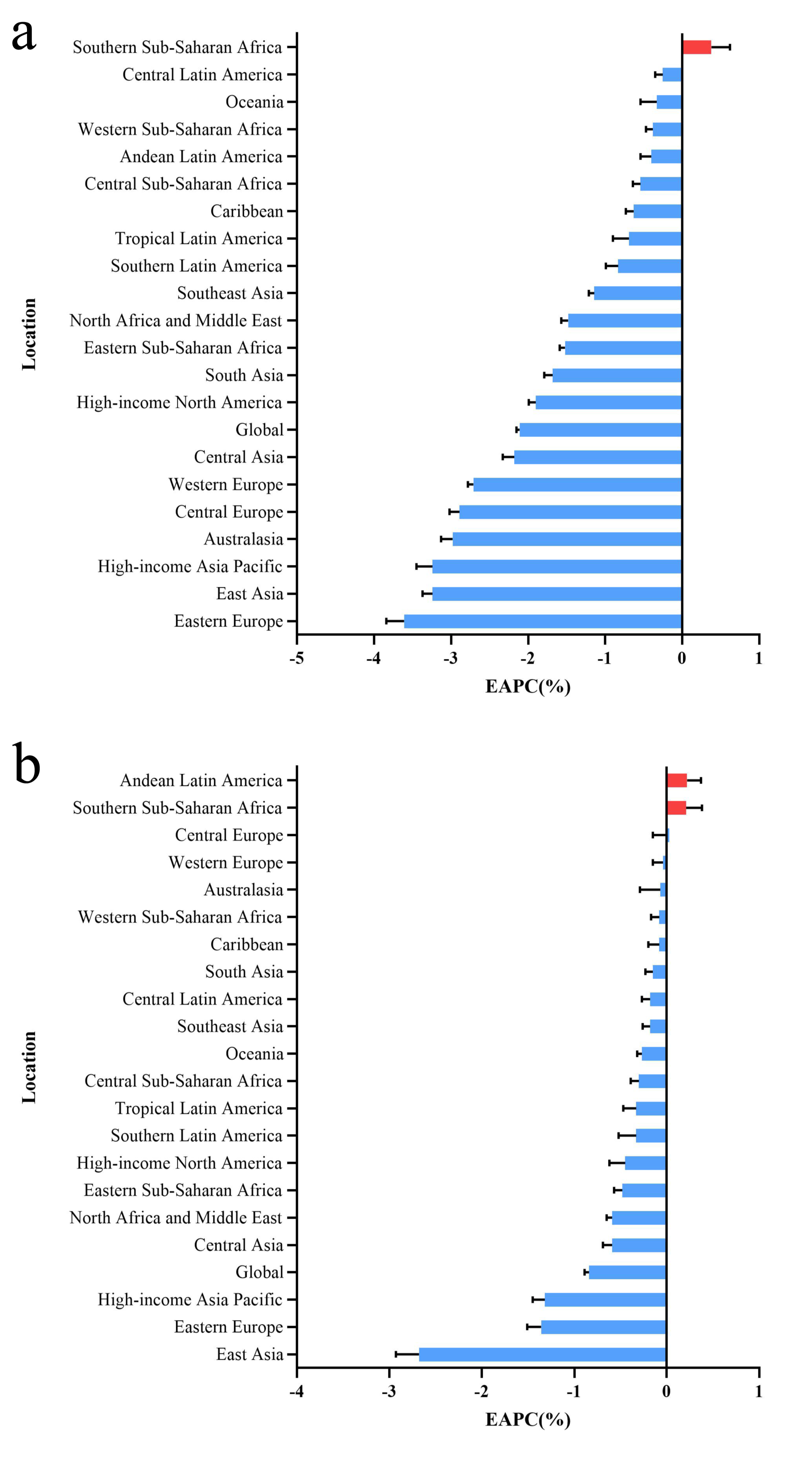

Supplement: S4 Fig — (a) ALL (b) AML. (TIF) [file pone.0330479.s004.tif]

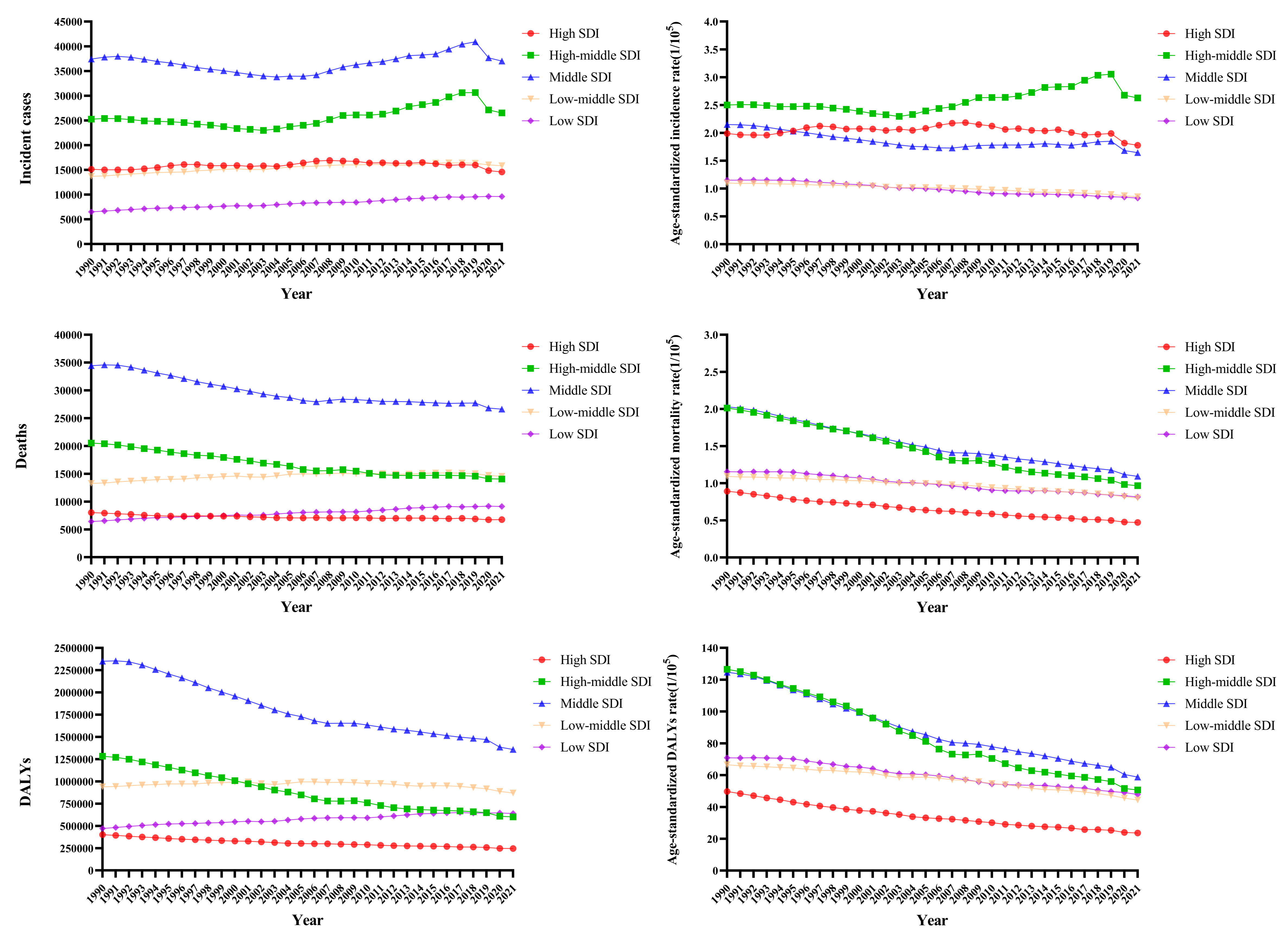

Supplement: S5 Fig — (TIF) [file pone.0330479.s005.tif]

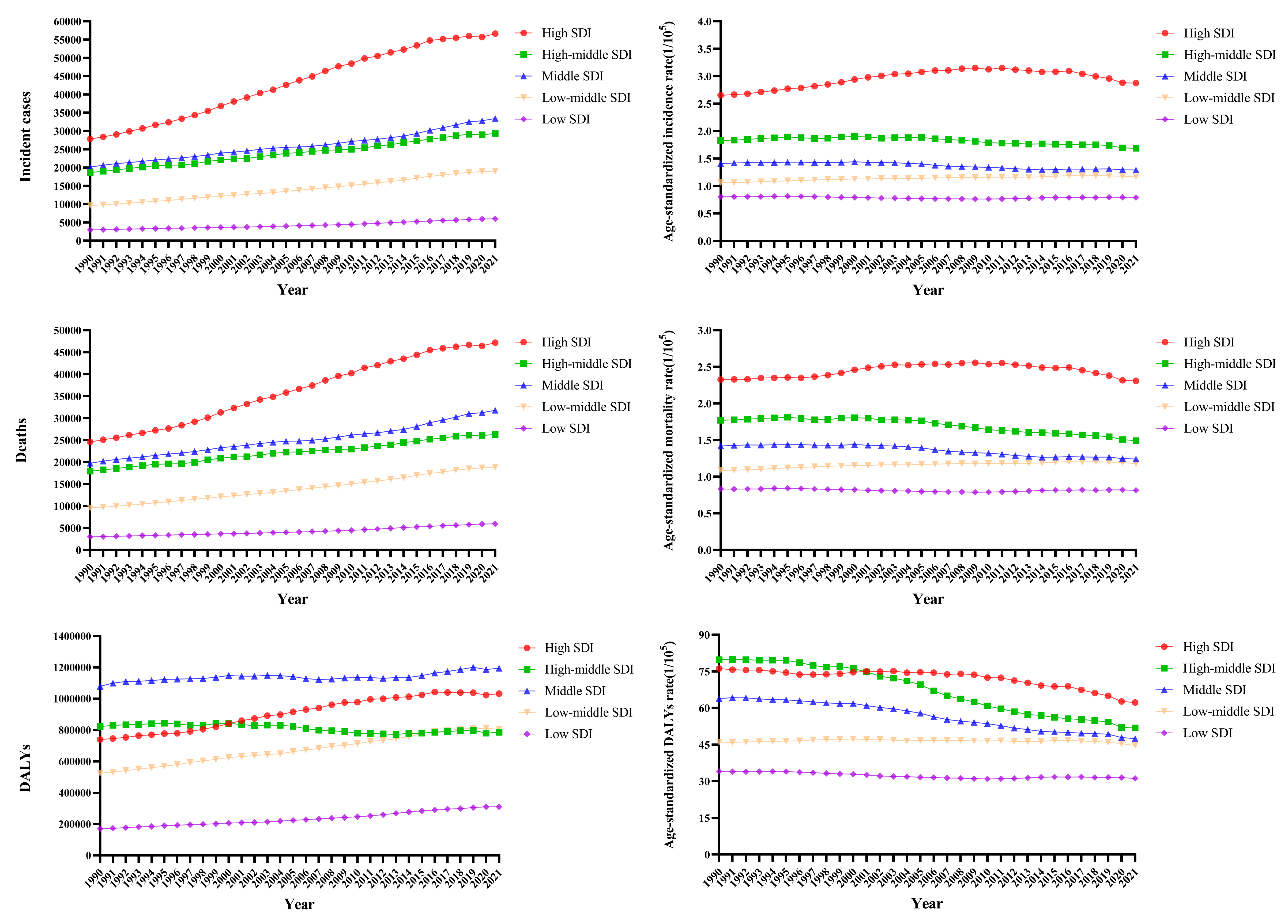

Supplement: S6 Fig — (TIF) [file pone.0330479.s006.tif]
