# Supplementary material for: Analysis of global, regional, and national burden and attributable risk factors of acute lymphoblastic leukemia and acute myeloid leukemia from 1990 to 2021
Source: PLoS One. 2025 Sep 2;20(9):e0330479. doi: 10.1371/journal.pone.0330479 (PMC12404455; doi:10.1371/journal.pone.0330479)
Supplement: S1 Table — (DOCX) [file pone.0330479.s007.docx]

**Supplementary Table 1 Regional incident cases and age-standardized incidence rate of acute leukemia in 2021**

| **REGION** | **Acute lymphoblastic leukemia** | | **Acute myeloid leukemia** | |
| --- | --- | --- | --- | --- |
|  | **Incident cases (×10^3^)** | **ASIR**^*^ | **Incident cases (×10^3^)** | **ASIR**^*^ |
| **Andean Latin America** | 1.45(0.95,1.84) | 2.26(1.49,2.88) | 1.07(0.74,1.34) | 1.72(1.20,2.17) |
| **Australasia** | 0.37(0.32,0.42) | 1.50(1.27,1.75) | 2.33(2.07,2.58) | 4.53(4.06,5.02) |
| **Caribbean** | 0.54(0.42,0.75) | 1.22(0.92,1.70) | 0.84(0.71,0.99) | 1.65(1.39,1.99) |
| **Central Asia** | 0.84(0.71,0.98) | 0.89(0.75,1.04) | 1.16(1.01,1.35) | 1.25(1.09,1.46) |
| **Central Europe** | 0.98(0.87, 1.10) | 0.91(0.79,1.05) | 4.23(3.85,4.59) | 2.11(1.92,2.29) |
| **Central Latin America** | 5.42(4.85,6.18) | 2.20(1.95,2.53) | 3.95(3.52,4.41) | 1.56(1.39,1.74) |
| **Central Sub-Saharan Africa** | 0.75(0.40,1.01) | 0.57(0.31,0.83) | 0.54(0.30,0.75) | 0.64(0.37,0.91) |
| **East Asia** | 39.30(21.67,51.65) | 3.57(1.97,4.96) | 19.16(13.18,26.30) | 1.07(0.74,1.48) |
| **Eastern Europe** | 2.09(1.94,2.24) | 1.08(1.00,1.16) | 4.28(3.92,4.65) | 1.43(1.32,1.55) |
| **Eastern Sub-Saharan Africa** | 4.49(2.86,6.10) | 1.01(0.63,1.35) | 1.88(1.01,2.67) | 0.63(0.36,0.89) |
| **High-income Asia Pacific** | 2.34(2.03,2.62) | 1.93(1.63,2.21) | 8.36(7.25,9.14) | 2.05(1.81,2.22) |
| **High-income North America** | 4.91(4.63,5.18) | 1.61(1.50,1.73) | 23.68(21.53,24.73) | 3.77(3.47,3.92) |
| **North Africa and Middle East** | 7.43(4.03,9.32) | 1.24(0.67,1.54) | 11.36(8.40,15.19) | 2.22(1.66,3.00) |
| **Oceania** | 0.05(0.03,0.09) | 0.36(0.19,0.60) | 0.20(0.10,0.28) | 1.85(0.95,2.63) |
| **South Asia** | 11.45(7.60,15.73) | 0.65(0.43,0.90) | 14.67(11.31,20.28) | 0.91(0.68,1.25) |
| **Southeast Asia** | 8.35(5.09,10.53) | 1.27(0.79,1.60) | 14.23(10.09,17.32) | 2.13(1.52,2.59) |
| **Southern Latin America** | 0.87(0.80,0.96) | 1.36(1.22,1.53) | 1.53(1.42,1.66) | 1.88(1.75,2.05) |
| **Southern Sub-Saharan Africa** | 0.64(0.37,0.80) | 0.84(0.49,1.06) | 0.89(0.61,1.24) | 1.37(0.92,1.88) |
| **Tropical Latin America** | 2.05(1.90,2.22) | 0.95(0.87,1.04) | 4.63(4.34,4.86) | 1.86(1.74,1.96) |
| **Western Europe** | 6.87(6.43,7.42) | 2.22(2.05,2.44) | 24.64(22.11,26.16) | 2.79(2.57,2.93) |
| **Western Sub-Saharan Africa** | 2.54(1.11,3.59) | 0.41(0.19,0.56) | 1.03(0.58,1.35) | 0.30(0.19,0.38) |

All data reported as number or rate (95% UI); ^*^Annual age-standardized rates (per 100,000 population)
