# Supplementary material for: Analysis of global, regional, and national burden and attributable risk factors of acute lymphoblastic leukemia and acute myeloid leukemia from 1990 to 2021
Source: PLoS One. 2025 Sep 2;20(9):e0330479. doi: 10.1371/journal.pone.0330479 (PMC12404455; doi:10.1371/journal.pone.0330479)
Supplement: S2 Table — (DOCX) [file pone.0330479.s008.docx]

**Supplementary Table 2 Regional deaths and age - standardized mortality rate of acute leukemia in 2021**

| **REGION** | **Acute lymphoblastic leukemia** | | **Acute myeloid leukemia** | |
| --- | --- | --- | --- | --- |
|  | **Deaths (×10^3^)** | **ASMR**^*^ | **Deaths (×10^3^)** | **ASMR**^*^ |
| **Andean Latin America** | 1.17(0.79,1.46) | 1.83(1.24,2.29) | 1.04(0.73,1.31) | 1.69(1.19,2.13) |
| **Australasia** | 0.15(0.13,0.16) | 0.38(0.35,0.41) | 1.52(1.36,1.67) | 2.82(2.55,3.09) |
| **Caribbean** | 0.49(0.37,0.70) | 1.08(0.80,1.56) | 0.81(0.68,0.96) | 1.58(1.32,1.92) |
| **Central Asia** | 0.69(0.58,0.80) | 0.74(0.63,0.85) | 1.08(0.94,1.27) | 1.19(1.04,1.39) |
| **Central Europe** | 0.78(0.70,0.86) | 0.51(0.46,0.57) | 3.94(3.59,4.28) | 1.91(1.74,2.08) |
| **Central Latin America** | 4.57(4.12,5.08) | 1.82(1.64,2.03) | 3.79(3.38,4.22) | 1.51(1.34,1.68) |
| **Central Sub-Saharan Africa** | 0.72(0.38,0.99) | 0.57(0.30,0.85) | 0.53(0.29,0.75) | 0.65(0.38,0.95) |
| **East Asia** | 21.21(12.23,27.79) | 1.35(0.77,1.73) | 16.51(11.57,22.72) | 0.91(0.64,1.27) |
| **Eastern Europe** | 1.65(1.52,1.78) | 0.64(0.59,0.68) | 3.91(3.59,4.27) | 1.28(1.18,1.39) |
| **Eastern Sub-Saharan Africa** | 4.23(2.71,5.71) | 0.98(0.62,1.31) | 1.86(0.99,2.67) | 0.64(0.37,0.91) |
| **High-income Asia Pacific** | 1.16(1.01,1.30) | 0.43(0.37,0.47) | 7.07(6.11,7.72) | 1.62(1.42,1.75) |
| **High-income North America** | 2.41(2.27,2.51) | 0.50(0.48,0.52) | 18.37(16.62,19.21) | 2.85(2.61,2.97) |
| **North Africa and Middle East** | 5.70(2.94,7.33) | 0.99(0.51,1.27) | 10.65(7.89,14.41) | 2.15(1.60,2.94) |
| **Oceania** | 0.05(0.03,0.09) | 0.36(0.20,0.57) | 0.20(0.10,0.28) | 1.91(0.97,2.73) |
| **South Asia** | 10.79(7.30,14.71) | 0.62(0.42,0.86) | 14.57(11.12,20.18) | 0.92(0.69,1.28) |
| **Southeast Asia** | 7.47(4.55,9.58) | 1.13(0.71,1.45) | 13.88(9.87,16.85) | 2.12(1.53,2.58) |
| **Southern Latin America** | 0.60(0.63,0.74) | 0.95(0.88,1.02) | 1.48(1.37,1.61) | 1.80(1.67,1.96) |
| **Southern Sub-Saharan Africa** | 0.61(0.35,0.77) | 0.82(0.47,1.06) | 0.88(0.60,1.21) | 1.39(0.94,1.92) |
| **Tropical Latin America** | 1.77(1.66,1.88) | 0.79(0.74,0.85) | 4.59(4.27,4.84) | 1.85(1.72,1.95) |
| **Western Europe** | 2.57(2.39,2.72) | 0.42(0.40,0.44) | 22.49(20.22,23.90) | 2.43(2.23,2.56) |
| **Western Sub-Saharan Africa** | 2.35(1.04,3.29) | 0.39(0.18,0.53) | 1.02(0.57,1.36) | 0.31(0.20,0.40) |

All data reported as number or rate (95% UI); ^*^Annual age-standardized rates (per 100,000 population
