# Supplementary material for: Analysis of global, regional, and national burden and attributable risk factors of acute lymphoblastic leukemia and acute myeloid leukemia from 1990 to 2021
Source: PLoS One. 2025 Sep 2;20(9):e0330479. doi: 10.1371/journal.pone.0330479 (PMC12404455; doi:10.1371/journal.pone.0330479)
Supplement: S3 Table — (DOCX) [file pone.0330479.s009.docx]

**Supplementary Table 3 Regional DALYs and age - standardized DALYs rate of acute leukemia in 2021**

| **REGION** | **Acute lymphoblastic leukemia** | | **Acute myeloid leukemia** | |
| --- | --- | --- | --- | --- |
|  | **DALYs (×10^3^)** | **ASDR**^*^ | **DALYs (×10^3^)** | **ASDR**^*^ |
| **Andean Latin America** | 65.18(44.43,82.89) | 99.87(67.97,127.17) | 40.59(27.53,50.64) | 63.28(42.95,79.01) |
| **Australasia** | 5.55(5.10,6.01) | 18.72(17.07,20.55) | 32.23(29.40,35.14) | 70.69(65.04,76.97) |
| **Caribbean** | 29.58(20.86,44.20) | 68.42(47.33,103.84) | 30.28(24.27,39.10) | 62.53(49.44,83.25) |
| **Central Asia** | 42.76(36.05,50.55) | 44.87(37.84,52.95) | 51.91(44.47,61.11) | 54.29(46.60,63.86) |
| **Central Europe** | 27.24(24.44,30.11) | 25.01(22.45,27.67) | 98.15(89.40,106.24) | 57.15(52.15,62.16) |
| **Central Latin America** | 252.73(226.92,284.86) | 101.43(90.60,115.35) | 148.60(131.94,167.20) | 58.45(51.73,65.95) |
| **Central Sub-Saharan Africa** | 48.32(26.41,67.10) | 30.97(16.46,43.22) | 27.16(14.71,37.58) | 24.25(13.50,34.31) |
| **East Asia** | 951.38(544.41,1214.73) | 73.59(42.79,94.11) | 586.47(412.97,820.00) | 38.00(26.53,53.49) |
| **Eastern Europe** | 61.20(56.77,65.88) | 30.47(28.50,32.63) | 121.07(111.22,131.58) | 46.75(43.27,50.48) |
| **Eastern Sub-Saharan Africa** | 301.93(193.92,414.82) | 58.47(37.02,79.19) | 106.79(56.64,150.40) | 26.61(14.25,38.29) |
| **High-income Asia Pacific** | 37.63(32.54,41.80) | 22.06(18.86,24.26) | 145.96(127.83,157.75) | 47.44(41.64,51.00) |
| **High-income North America** | 87.72(84.28,91.05) | 23.80(22.83,24.84) | 402.42(377.76,415.93) | 72.60(68.88,74.87) |
| **North Africa and Middle East** | 338.00(181.11,432.53) | 54.14(28.94,69.27) | 422.23(309.21,574.87) | 73.97(54.53,100.93) |
| **Oceania** | 3.69(1.86,6.49) | 22.12(11.11,38.27) | 10.74(5.84,14.80) | 79.86(42.08,111.92) |
| **South Asia** | 623.01(432.19,830.91) | 34.07(23.54,45.32) | 619.34(460.68,830.68) | 35.44(26.75,47.89) |
| **Southeast Asia** | 411.94(259.34,504.35) | 62.21(39.61,75.73) | 537.64(379.22,664.18) | 77.30(54.85,96.68) |
| **Southern Latin America** | 31.68(29.48,34.33) | 49.12(45.54,53.67) | 45.34(42.35,49.08) | 60.80(56.76,65.99) |
| **Southern Sub-Saharan Africa** | 34.27(21.32,43.16) | 42.52(26.03,53.51) | 34.64(23.60,49.75) | 47.56(32.42,67.27) |
| **Tropical Latin America** | 93.87(86.28,101.53) | 44.86(40.55,49.17) | 152.69(145.51,159.60) | 63.07(60.06,66.06) |
| **Western Europe** | 88.65(84.61,93.12) | 21.76(20.74,23.13) | 460.400(427.28,482.07) | 63.60(60.57,66.12) |
| **Western Sub-Saharan Africa** | 183.46(78.70,259.03) | 26.90(11.98,37.55) | 60.40(31.37,85.65) | 12.32(7.00,16.31) |

All data reported as number or rate (95% UI); ^*^Annual age-standardized rates (per 100,000 population)
