# Supplementary material for: Analysis of global, regional, and national burden and attributable risk factors of acute lymphoblastic leukemia and acute myeloid leukemia from 1990 to 2021
Source: PLoS One. 2025 Sep 2;20(9):e0330479. doi: 10.1371/journal.pone.0330479 (PMC12404455; doi:10.1371/journal.pone.0330479)
Supplement: S4 Table — (DOCX) [file pone.0330479.s010.docx]

**Supplementary Table 4 Incidence, mortality and DALYs of acute leukemia among the top three and bottom three countries in 2021**

| Measure | Type | Sex | Top three countries | | | | | | | | | | Bottom three countries | | | | | | | | | | | | | |
| --- | --- | --- | --- | --- | --- | --- | --- | --- | --- | --- | --- | --- | --- | --- | --- | --- | --- | --- | --- | --- | --- | --- | --- | --- | --- | --- |
| Incident cases | Acute lymphoblastic leukemia | Both | China  (38570.94) | | India  (7751.12) | | | | USA  (4368.58) | | | | Cook Islands  (0.02) | | Niue  (0.01) | | | | | | | | | | Tokelau  (0.01) | |
|  |  | Male | China  (23183.20) | | India  (5038.29) | | | | USA  (2387.32) | | Northern Mariana  Islands (0.00) | | | | American Samoa  (0.00) | | | | | | | | | | Bermuda  (0.00) | |
|  |  | Female | China  (15387.75) | | India  (2712.83) | | | | USA  (1981.26) | | | | Niue  (0.01) | | Tokelau  (0.01) | | | | | | | | | | Seychelles (0.00) | |
|  | Acute myeloid leukemia | Both | USA  (21533.02) | | China  (17835.21) | | | | India  (11040.27) | | | | Palau  (0.08) | | Niue  (0.05) | | | | | | | | | | Tokelau  (0.05) | |
|  |  | Male | USA  (12114.98) | | China  (9879.80) | | | | India  (5682.13) | | | | Niue  (0.03) | | Palau  (0.03) | | | | | | | | | | Tokelau  (0.03) | |
|  |  | Female | USA  (9418.04) | | China  (7955.41) | | | | India  (5358.14) | | | | Niue  (0.02) | | Tokelau  (0.02) | | | | | Saint Kitts and  Nevis (0.00) | | | | | | |
| Deaths | Acute lymphoblastic leukemia | Both | China  (20612.91) | | India  (7323.35) | | | | Indonesia (3163.31) | | | | Cook Islands  (0.01) | | Niue  (0.01) | | | | | | | | | | Tokelau  (0.01) | |
|  |  | Male | China  (12479.97) | | India  (4751.59) | | | | Indonesia  (2070.68) | | Northern Mariana  Islands (0.00) | | | | American Samoa  (0.00) | | | | | | | | | | Bermuda  (0.00) | |
|  |  | Female | China  (8132.94) | | India  (2571.77) | | | | Mexico  (1231.50) | | | | Niue  (0.01) | | Tokelau  (0.01) | | | | | | | | | | Seychelles (0.00) | |
|  | Acute myeloid leukemia | Both | USA  (16648.46) | | China  (15311.11) | | | | India  (10981.08) | | | | Palau  (0.08) | | Niue  (0.05) | | | | | | | | | | Tokelau  (0.05) | |
|  |  | Male | USA  (9616.495) | | China  (8691.51) | | | | India  (5697.95) | | | | Niue  (0.03) | | Palau  (0.03) | | | | | | | | | | Tokelau  (0.03) | |
|  |  | Female | USA  (7031.97) | | China  (6619.60) | | | | India  (5283.13) | | | | Niue  (0.02) | | Tokelau  (0.02) | | | | | Saint Kitts and  Nevis (0.00) | | | | | | |
| DALYs | Acute lymphoblastic leukemia | Both | China  (924422.19) | | India  (396018.03) | | | | Indonesia  (175191.51) | | | | Tokelau  (0.77) | | Niue  (0.75) | | | | | | | | Cook Islands  (0.52) | | | |
|  |  | Male | China  (572259.10) | | India  (256716.32) | | | | Indonesia  (116353.14) | | | Northern Mariana  Islands (0.02) | | | | American Samoa  (0.01) | | | | | | | | | | Bermuda  (0.00) |
|  |  | Female | China  (352163.09) | | India  (139301.71) | | | | Ethiopia  (66095.31) | | | | Tokelau  (0.67) | | Cook Islands  (0.42) | | | | | | | | | | Seychelles  (0.01) | |
|  | Acute myeloid leukemia | Both | China  (548555.39) | | India  (439739.96) | | | | USA  (366031.69) | | | | Palau  (3.43) | | Tokelau  (2.70) | | | | | | | | | | Niue  (2.56) | |
|  |  | Male | China  (313668.38) | | India  (220544.18) | | | | USA  (211446.35) | | | | Tokelau  (1.60) | | Niue  (1.47) | | | | | | | | | | Palau  (1.16) | |
|  |  | Female | China  (234887.01) | | India  (219195.78) | | | | USA  (154585.34) | | | | Tokelau  (1.10) | | Niue  (1.09) | | | | | | | Saint Kitts and  Nevis (0.00) | | | | |
| ASIR^*^ | Acute lymphoblastic leukemia | Both | Monaco  (8.81) | | San Marino  (5.98) | | | | China  (3.64) | | | | Tonga  (0.18) | | Guinea  (0.16) | | | | | | | | Cook Islands  (0.14) | | | |
|  |  | Male | Monaco  (8.46) | | San Marino  (5.99) | | | | China  (4.20) | | | | American Samoa  (0.00) | | | | | Oman  (0.00) | | | | | | | Bermuda  (0.00) | |
|  |  | Female | Monaco  (9.16) | | San Marino  (5.98) | | | | Afghanistan  (4.06) | | | | Gambia  (0.16) | | Guinea  (0.14) | | | | | | | | | | Seychelles  (0.00) | |
|  | Acute myeloid leukemia | Both | Monaco  (6.30) | | Australia  (4.92) | | | | Fiji  (4.15) | | | | Malawi  (0.16) | | Gambia  (0.15) | | | | | | | | | | Guinea  (0.11) | |
|  |  | Male | Monaco  (6.73) | | Australia  (6.29) | | | | Fiji  (5.70) | | | | Mali  (0.13) | | Sao Tome and  Principe (0.10) | | | | | | | | | | Guinea  (0.09) | |
|  |  | Female | Monaco  (6.73) | United Arab  Emirates (5.59) | | | | | Afghanistan  (4.78) | | | | Malawi  (0.14) | | Guinea  (0.12) | | | | | | Saint Kitts and  Nevis (0.00) | | | | | |
| ASMR^*^ | Acute lymphoblastic leukemia | Both | Afghanistan  (2.94) | Bolivia (Plurinational  State of) (2.24) | | | | | | Ecuador  (2.16) | | | Bermuda  (0.15) | | Oman  (0.11) | | | | | | | | Cook Islands  (0.08) | | | |
|  |  | Male | Dominica  (2.60) | | Mexico  (2.25) | | | | Ecuador  (2.21) | | | | American Samoa  (0.00) | | | | Oman  (0.00) | | | | | | | | Bermuda (0.00) | |
|  |  | Female | Afghanistan  (4.09) | | Haiti  (2.44) | | Bolivia (Plurinational  State of) (2.29) | | | | | | Guinea  (0.13) | | Cook Islands  (0.13) | | | | | | | | | | Seychelles (0.00) | |
|  | Acute myeloid leukemia | Both | Monaco  (5.34) | | Fiji  (4.27) | | | | Afghanistan  (3.94) | | | | Gambia  (0.16) | | Malawi  (0.16) | | | | | | | | | | Guinea  (0.11) | |
|  |  | Male | Fiji  (6.12) | | Monaco  (5.98) | | | | Jordan  (4.29) | | | | Mali  (0.14) | | Sao Tome and  Principe (0.11) | | | | | | | | | | Guinea  (0.10) | |
|  |  | Female | United Arab  Emirates (5.71) | | | Afghanistan  (4.84) | | | Monaco  (4.81) | | | | Malawi  (0.14) | | Guinea  (0.12) | | | | | | Saint Kitts and Nevis (0.00) | | | | | |
| ASDR^*^ | Acute lymphoblastic leukemia | Both | Afghanistan  (148.05) | Bolivia (Plurinational  State of) (122.80) | | | | | | Haiti  (115.62) | | | American Samoa  (7.77) | | | | Oman  (5.45) | | | | | | | Cook Islands  (3.34) | | |
|  |  | Male | Dominica  (137.60) | | Mexico  (127.72) | | Bolivia (Plurinational  State of) (125.52) | | | | | | American Samoa  (0.05) | | | | | | Oman  (0.00) | | | | | | Bermuda (0.00) | |
|  |  | Female | Afghanistan  (200.57) | | Haiti  (156.17) | | | Bolivia (Plurinational  State of) (119.52) | | | | | Qatar  (7.29) | | Cook Islands  (5.25) | | | | | | | | | | Seychelles (0.01) | |
|  | Acute myeloid leukemia | Both | Tokelau  (204.88) | | Niue  (169.15) | | | | Fiji  (167.10) | | | | Malawi  (7.08) | | Gambia  (5.97) | | | | | | | | | | Guinea  (4.62) | |
|  |  | Male | Tokelau  (239.15) | | Fiji  (204.33) | | | | Niue  (187.49) | | | | Gambia  (4.85) | | Guinea  (3.81) | | | | | | Sao Tome and  Principe (3.74) | | | | | |
|  |  | Female | Tokelau  (169.70) | | Afghanistan  (168.96) | | | | Niue  (153.73) | | | | Guinea  (5.41) | Northern Mariana  Islands (4.97) | | | | | | | Saint Kitts and Nevis (0.00) | | | | | |

^*^Annual age-standardized rates (per 100,000 population)
