# Supplementary material for: Analysis of global, regional, and national burden and attributable risk factors of acute lymphoblastic leukemia and acute myeloid leukemia from 1990 to 2021
Source: PLoS One. 2025 Sep 2;20(9):e0330479. doi: 10.1371/journal.pone.0330479 (PMC12404455; doi:10.1371/journal.pone.0330479)
Supplement: S5 Table — (DOCX) [file pone.0330479.s011.docx]

**Supplementary Table 5 EAPC of ASIR for acute leukemia in 204 countries and territories from 1990 to 2021**

| Location | EAPC (95%*CI*) | |
| --- | --- | --- |
|  | Acute lymphoblastic leukemia | Acute myeloid leukemia |
| Afghanistan | -0.40% (-0.50, -0.30) | 0.31% (0.25, 0.38) |
| Albania | 0.25% (-0.05, 0.55) | -0.06% (-0.22, 0.10) |
| Algeria | -0.74% (-0.83, -0.65) | -0.28% (-0.35, -0.21) |
| American Samoa | 0.93% (0.36, 1.52) | -2.84% (-3.19, -2.48) |
| Andorra | -1.28% (-1.70, -0.86) | -0.67% (-0.85, -0.49) |
| Angola | -0.82% (-0.91, -0.73) | -0.16% (-0.23, -0.09) |
| Antigua and Barbuda | 0.25% (-0.13, 0.63) | 1.17% (0.93, 1.40) |
| Argentina | 0.18% (-0.01, 0.38) | 0.30% (0.02, 0.57) |
| Armenia | -2.59% (-2.95, -2.22) | 2.05% (1.33, 2.78) |
| Australia | 0.21% (-0.22, 0.64) | 1.14% (0.82, 1.47) |
| Austria | -0.36% (-0.74, 0.01) | 1.19% (0.93, 1.45) |
| Azerbaijan | -1.27% (-1.47, -1.06) | -0.50% (-0.64, -0.36) |
| Bahamas | -0.40% (-0.70, -0.11) | 1.02% (0.74, 1.31) |
| Bahrain | -0.90% (-1.04, -0.76) | -1.49% (-1.69, -1.29) |
| Bangladesh | -1.30% (-1.39, -1.21) | -0.39% (-0.46, -0.33) |
| Barbados | -0.73% (-1.11, -0.35) | 1.42% (1.23, 1.62) |
| Belarus | -2.41% (-2.78, -2.03) | 1.53% (1.00, 2.06) |
| Belgium | -0.90% (-1.19, -0.60) | 1.00% (0.71, 1.29) |
| Belize | -0.69% (-1.02, -0.36) | 1.72% (1.33, 2.11) |
| Benin | 0.56% (0.41, 0.71) | 0.65% (0.55, 0.74) |
| Bermuda | 2.25% (1.98, 2.51) | -0.58% (-0.75, -0.41) |
| Bhutan | -0.86% (-1.02, -0.70) | 0.05% (-0.03, 0.13) |
| Bolivia (Plurinational State of) | -0.89% (-0.94, -0.85) | 0.18% (0.15, 0.21) |
| Bosnia and Herzegovina | -0.35% (-0.54, -0.16) | 0.61% (0.47, 0.76) |
| Botswana | -0.34% (-0.51, -0.17) | -0.19% (-0.32, -0.06) |
| Brazil | -0.05% (-0.23, 0.13) | 0.28% (0.15, 0.40) |
| Brunei Darussalam | -0.89% (-1.07, -0.70) | -0.40% (-0.53, -0.26) |
| Bulgaria | -1.62% (-1.99, -1.23) | 2.36% (2.02, 2.70) |
| Burkina Faso | 0.87% (0.71, 1.03) | 0.98% (0.88, 1.09) |
| Burundi | -0.60% (-0.73, -0.46) | -0.42% (-0.50, -0.33) |
| Cabo Verde | -0.42% (-0.59, -0.25) | 0.36% (0.10, 0.62) |
| Cambodia | -1.13% (-1.19, -1.06) | -0.04% (-0.08, 0) |
| Cameroon | 0.75% (0.56, 0.94) | 0.66% (0.56, 0.76) |
| Canada | -0.59% (-0.91, -0.27) | 1.06% (0.86, 1.26) |
| Central African Republic | -0.28% (-0.33, -0.22) | -0.36% (-0.40, -0.32) |
| Chad | 1.50% (1.34, 1.66) | 1.45% (1.32, 1.58) |
| Chile | 0.85% (0.66, 1.04) | 0.68% (0.49, 0.87) |
| China | 0.63% (0.34, 0.92) | -1.51% (-1.70, -1.32) |
| Colombia | 1.17% (0.85, 1.50) | 0.66% (0.49, 0.84) |
| Comoros | -0.33% (-0.62, -0.04) | -0.16% (-0.36, 0.04) |
| Congo | -0.54% (-0.68, -0.39) | -0.55% (-0.66, -0.45) |
| Cook Islands | -1.77% (-2.24, -1.30) | -0.76% (-0.86, -0.67) |
| Costa Rica | 1.48% (1.30, 1.66) | 1.11% (0.88, 1.34) |
| Côte d'Ivoire | 0.04% (-0.11, 0.19) | -0.25% (-0.35, -0.15) |
| Croatia | -0.01% (-0.31, 0.28) | 1.77% (1.60, 1.94) |
| Cuba | -0.35% (-0.57, -0.13) | 0.17% (-0.03, 0.37) |
| Cyprus | 2.17% (1.56, 2.78) | 0.07% (-0.11, 0.25) |
| Czechia | -0.96% (-1.22, -0.7) | 0.81% (0.55, 1.07) |
| Democratic People's Republic of Korea | 0.04% (-0.11, 0.19) | -0.57% (-0.61, -0.54) |
| Democratic Republic of the Congo | -0.12% (-0.22, -0.03) | -0.22% (-0.36, -0.09) |
| Denmark | 0.80% (0.54, 1.06) | -0.51% (-0.73, -0.29) |
| Djibouti | 0% (-0.26, 0.26) | 0.21% (0.07, 0.35) |
| Dominica | 0.95% (0.82, 1.09) | 0.22% (0.15, 0.28) |
| Dominican Republic | -0.80% (-0.90, -0.70) | 0.60% (0.46, 0.73) |
| Ecuador | 1.60% (1.28, 1.92) | 1.88% (1.42, 2.35) |
| Egypt | 1.17% (0.82, 1.53) | 2.08% (1.78, 2.39) |
| El Salvador | 0.15% (0.01, 0.29) | 0.74% (0.64, 0.84) |
| Equatorial Guinea | -1.52% (-1.75, -1.29) | -0.11% (-0.20, -0.01) |
| Eritrea | 0.12% (0.01, 0.23) | 0.36% (0.28, 0.45) |
| Estonia | -1.45% (-1.75, -1.15) | 1.00% (0.52, 1.48) |
| Eswatini | 0.85% (0.49, 1.21) | 0.77% (0.39, 1.15) |
| Ethiopia | -2.12% (-2.29, -1.96) | -0.92% (-1.06, -0.78) |
| Fiji | -1.2% (-1.52, -0.88) | -0.08% (-0.25, 0.10) |
| Finland | 1.38% (1.04, 1.73) | 0.37% (0.22, 0.51) |
| France | 0.68% (0.48, 0.88) | 1.21% (1.01, 1.41) |
| Gabon | -0.07% (-0.19, 0.05) | -0.28% (-0.37, -0.19) |
| Gambia | -0.66% (-0.91, -0.42) | -0.10% (-0.29, 0.10) |
| Georgia | -2.91% (-3.49, -2.32) | 2.31% (1.57, 3.07) |
| Germany | 0.08% (-0.28, 0.44) | 1.12% (1.02, 1.22) |
| Ghana | -2.99% (-3.49, -2.49) | -2.16% (-2.60, -1.71) |
| Greece | -0.74% (-1.00, -0.48) | 1.21% (1.06, 1.36) |
| Greenland | -2.53% (-2.71, -2.36) | -1.12% (-1.19, -1.05) |
| Grenada | -0.42% (-0.69, -0.16) | 1.08% (0.94, 1.23) |
| Guam | -0.89% (-1.70, -0.07) | 0.33% (-0.14, 0.80) |
| Guatemala | 1.57% (1.34, 1.81) | 0.72% (0.48, 0.96) |
| Guinea | -0.60% (-0.73, -0.48) | -0.24% (-0.28, -0.20) |
| Guinea-Bissau | 0.16% (-0.07, 0.40) | 0.77% (0.65, 0.89) |
| Guyana | 1.50% (1.03, 1.97) | 1.91% (1.66, 2.16) |
| Haiti | -0.62% (-0.72, -0.51) | -0.05% (-0.11, 0.02) |
| Honduras | -1.13% (-1.24, -1.02) | 0.14% (0.08, 0.20) |
| Hungary | -0.77% (-0.99, -0.55) | 0.69% (0.54, 0.84) |
| Iceland | -0.14% (-0.39, 0.11) | 0.73% (0.64, 0.83) |
| India | -1.54% (-1.64, -1.44) | 0.17% (0.07, 0.27) |
| Indonesia | -0.45% (-0.55, -0.35) | 0.22% (0.12, 0.33) |
| Iran (Islamic Republic of) | -0.62% (-0.84, -0.41) | -0.37% (-0.49, -0.26) |
| Iraq | -0.41% (-0.49, -0.33) | 0.04% (-0.05, 0.13) |
| Ireland | 0.11% (-0.39, 0.61) | 0.51% (0.21, 0.81) |
| Israel | -0.09% (-0.45, 0.27) | 0.49% (0.25, 0.73) |
| Italy | -1.08% (-1.50, -0.67) | 1.28% (1.03, 1.53) |
| Jamaica | -0.86% (-1.11, -0.62) | 2.39% (2.02, 2.76) |
| Japan | -0.72% (-0.96, -0.48) | -0.06% (-0.30, 0.19) |
| Jordan | -1.12% (-1.34, -0.91) | -1.14% (-1.34, -0.93) |
| Kazakhstan | -1.41% (-1.64, -1.19) | -0.56% (-0.88, -0.25) |
| Kenya | 0.53% (0.32, 0.74) | 1.08% (0.95, 1.21) |
| Kiribati | -0.21% (-0.35, -0.06) | 0.15% (0.08, 0.22) |
| Kuwait | -0.63% (-0.92, -0.34) | -0.34% (-0.89, 0.22) |
| Kyrgyzstan | -2.15% (-2.51, -1.78) | 1.64% (1.31, 1.96) |
| Lao People's Democratic Republic | -1.47% (-1.55, -1.39) | -0.36% (-0.41, -0.30) |
| Latvia | -2.27% (-2.42, -2.12) | -0.60% (-0.98, -0.21) |
| Lebanon | 0.12% (0.03, 0.20) | 0.07% (-0.05, 0.19) |
| Lesotho | 2.34% (1.99, 2.70) | 2.03% (1.76, 2.3) |
| Liberia | 0.37% (-0.01, 0.75) | 0.9% (0.68, 1.13) |
| Libya | 1.72% (1.45, 1.99) | 0.18% (0.05, 0.31) |
| Lithuania | -1.98% (-2.22, -1.74) | 2.18% (1.81, 2.55) |
| Luxembourg | -1.45% (-1.78, -1.13) | 0.57% (0.36, 0.78) |
| Madagascar | -0.21% (-0.30, -0.12) | -0.27% (-0.38, -0.16) |
| Malawi | -1.24% (-1.33, -1.14) | -0.61% (-0.70, -0.53) |
| Malaysia | -0.77% (-0.95, -0.58) | -0.02% (-0.14, 0.11) |
| Maldives | -1.15% (-1.33, -0.97) | -1.54% (-1.6, -1.48) |
| Mali | -0.96% (-1.07, -0.85) | -0.52% (-0.58, -0.46) |
| Malta | 1.14% (0.89, 1.39) | 0.93% (0.7, 1.16) |
| Marshall Islands | 0.09% (-0.19, 0.37) | 0.34% (0.27, 0.4) |
| Mauritania | 0.27% (0.11, 0.43) | 0.52% (0.42, 0.61) |
| Mauritius | 2.57% (-1.01, 6.28) | 5.12% (1.91, 8.43) |
| Mexico | 0.58% (0.37, 0.79) | 0.19% (0.08, 0.29) |
| Micronesia (Federated States of) | -0.64% (-0.72, -0.56) | -0.19% (-0.22, -0.16) |
| Monaco | -1.02% (-1.47, -0.57) | 1.16% (0.94, 1.38) |
| Mongolia | -1.69% (-1.91, -1.47) | 0% (-0.11, 0.11) |
| Montenegro | -0.88% (-1.14, -0.63) | 0.32% (0.22, 0.42) |
| Morocco | -0.86% (-0.95, -0.77) | 0.23% (0.18, 0.28) |
| Mozambique | -0.67% (-0.83, -0.52) | 0.09% (0.01, 0.18) |
| Myanmar | -1.72% (-1.87, -1.58) | -0.73% (-0.84, -0.63) |
| Namibia | 0.04% (-0.07, 0.14) | 0.03% (-0.14, 0.21) |
| Nauru | 0.19% (-0.14, 0.52) | -0.38% (-0.45, -0.32) |
| Nepal | -1.1% (-1.23, -0.98) | 0.31% (0.15, 0.46) |
| Netherlands | -1.36% (-1.83, -0.89) | 0.55% (0.37, 0.74) |
| New Zealand | 0.09% (-0.31, 0.50) | -0.57% (-0.93, -0.21) |
| Nicaragua | -0.47% (-0.67, -0.27) | 0.27% (0.12, 0.43) |
| Niger | -0.29% (-0.44, -0.14) | 0.48% (0.40, 0.56) |
| Nigeria | -0.11% (-0.20, -0.02) | 0.07% (-0.01, 0.14) |
| Niue | 1.17% (0.32, 2.02) | 0.32% (-0.10, 0.73) |
| North Macedonia | -0.78% (-1.05, -0.52) | -0.15% (-0.35, 0.06) |
| Northern Mariana Islands | -1.5% (-1.73, -1.26) | -3.23% (-3.53, -2.93) |
| Norway | 0.33% (-0.04, 0.70) | 0% (-0.13, 0.14) |
| Oman | -0.31% (-0.63, 0.01) | -0.45% (-0.62, -0.27) |
| Pakistan | -0.17% (-0.28, -0.06) | 0.30% (0.18, 0.42) |
| Palau | -0.18% (-0.31, -0.06) | -0.06% (-0.12, 0) |
| Palestine | -0.78% (-0.90, -0.65) | -0.62% (-0.7, -0.54) |
| Panama | 1.09% (0.97, 1.22) | 1.16% (0.99, 1.33) |
| Papua New Guinea | -0.28% (-0.48, -0.08) | -0.06% (-0.1, -0.01) |
| Paraguay | 0.47% (0.25, 0.70) | 1.12% (0.93, 1.30) |
| Peru | 0.88% (0.72, 1.05) | 0.48% (0.34, 0.62) |
| Philippines | -0.58% (-0.67, -0.49) | -0.25% (-0.33, -0.18) |
| Poland | -0.92% (-1.19, -0.64) | 0.74% (0.43, 1.04) |
| Portugal | 0.09% (-0.19, 0.37) | 0.75% (0.56, 0.94) |
| Puerto Rico | -0.17% (-0.42, 0.07) | 0.11% (-0.08, 0.30) |
| Qatar | -0.24% (-0.37, -0.11) | -1.63% (-1.93, -1.33) |
| Republic of Korea | 1.47% (1.12, 1.82) | -0.07% (-0.22, 0.08) |
| Republic of Moldova | -2.26% (-2.48, -2.04) | -0.30% (-0.77, 0.18) |
| Romania | -0.91% (-1.13, -0.69) | 1.62% (1.48, 1.77) |
| Russian Federation | -1.48% (-1.84, -1.11) | 0.26% (0.04, 0.48) |
| Rwanda | -1.66% (-1.82, -1.49) | -1.03% (-1.18, -0.88) |
| Saint Kitts and Nevis | -0.38% (-0.62, -0.14) | 1.12% (0.90, 1.34) |
| Saint Lucia | -0.61% (-0.88, -0.35) | 0.08% (-0.10, 0.27) |
| Saint Vincent and the Grenadines | -0.30% (-0.66, 0.07) | 0.78% (0.61, 0.95) |
| Samoa | -0.02% (-0.06, 0.02) | -0.24% (-0.33, -0.15) |
| San Marino | -1.47% (-1.69, -1.25) | -0.68% (-1.01, -0.35) |
| Sao Tome and Principe | -1.63% (-1.81, -1.46) | -0.27% (-0.40, -0.15) |
| Saudi Arabia | 0.36% (0.14, 0.58) | 1.49% (1.10, 1.89) |
| Senegal | 0.18% (-0.06, 0.42) | 0.95% (0.79, 1.11) |
| Serbia | -1.78% (-1.98, -1.57) | 0.21% (0.12, 0.30) |
| Seychelles | -0.42% (-0.74, -0.10) | -0.37% (-0.48, -0.25) |
| Sierra Leone | 0.66% (0.47, 0.84) | 1.10% (0.95, 1.25) |
| Singapore | 1.31% (0.96, 1.67) | -0.21% (-0.49, 0.08) |
| Slovakia | -0.37% (-0.48, -0.26) | -0.05% (-0.14, 0.03) |
| Slovenia | 0.20% (-0.15, 0.56) | 1.41% (1.14, 1.67) |
| Solomon Islands | 0.07% (-0.05, 0.19) | 0.23% (0.16, 0.30) |
| Somalia | 0.15% (-0.04, 0.33) | -0.03% (-0.10, 0.04) |
| South Africa | -0.02% (-0.22, 0.19) | 0.16% (0.03, 0.29) |
| South Sudan | 0.82% (0.45, 1.18) | 0.18% (0.02, 0.34) |
| Spain | -0.57% (-0.96, -0.18) | 1.00% (0.83, 1.17) |
| Sri Lanka | -1.47% (-1.67, -1.27) | -0.79% (-0.95, -0.63) |
| Sudan | -0.73% (-0.8, -0.67) | 0.17% (0.11, 0.23) |
| Suriname | -0.37% (-0.58, -0.16) | 0.51% (0.37, 0.65) |
| Sweden | -1.73% (-2.12, -1.34) | 0.11% (-0.06, 0.28) |
| Switzerland | -2.07% (-2.33, -1.81) | 0.52% (0.30, 0.73) |
| Syrian Arab Republic | -1.17% (-1.47, -0.87) | -0.75% (-0.90, -0.59) |
| Taiwan (Province of China) | 2.42% (2.07, 2.77) | 1.40% (1.11, 1.70) |
| Tajikistan | -1.77% (-1.99, -1.56) | -0.90% (-1.04, -0.76) |
| Thailand | -0.43% (-0.57, -0.30) | 0.78% (0.68, 0.88) |
| Timor-Leste | -0.93% (-1.20, -0.67) | 0.04% (-0.18, 0.25) |
| Togo | 0.57% (0.44, 0.71) | 1.01% (0.92, 1.09) |
| Tokelau | 0.45% (-0.72, 1.63) | 0.32% (-0.30, 0.94) |
| Tonga | 0.02% (-0.19, 0.24) | 0.38% (0.27, 0.49) |
| Trinidad and Tobago | -0.42% (-0.6, -0.23) | 0.78% (0.66, 0.90) |
| Tunisia | -0.65% (-0.75, -0.54) | -0.32% (-0.35, -0.29) |
| Türkiye | -0.98% (-1.10, -0.86) | -1.15% (-1.37, -0.93) |
| Turkmenistan | -1.19% (-1.48, -0.90) | 0.07% (-0.22, 0.36) |
| Tuvalu | -1.13% (-1.18, -1.07) | -0.09% (-0.12, -0.07) |
| Uganda | -0.30% (-0.44, -0.15) | 0.40% (0.28, 0.51) |
| Ukraine | -2.55% (-2.82, -2.29) | -2.20% (-2.45, -1.95) |
| United Arab Emirates | -1.24% (-1.50, -0.97) | 0.13% (-0.20, 0.46) |
| United Kingdom | -0.84% (-1.19, -0.50) | 0.26% (0.12, 0.39) |
| United Republic of Tanzania | -0.11% (-0.26, 0.03) | -0.02% (-0.07, 0.02) |
| United States of America | -0.78% (-0.98, -0.57) | 0.37% (0.14, 0.61) |
| United States Virgin Islands | -0.76% (-0.98, -0.55) | -0.61% (-0.79, -0.43) |
| Uruguay | -0.04% (-0.25, 0.17) | 0.38% (0.24, 0.52) |
| Uzbekistan | -1.52% (-1.79, -1.26) | -0.58% (-0.9, -0.26) |
| Vanuatu | 0.03% (-0.19, 0.24) | 0.04% (-0.02, 0.10) |
| Venezuela (Bolivarian Republic of) | 0.80% (0.54, 1.06) | 0.65% (0.48, 0.82) |
| Viet Nam | -0.47% (-0.57, -0.38) | -0.26% (-0.35, -0.18) |
| Yemen | -0.73% (-0.83, -0.62) | 0.19% (0.12, 0.27) |
| Zambia | -1.44% (-1.65, -1.22) | 0.61% (0.50, 0.72) |
| Zimbabwe | 2.23% (1.75, 2.71) | 1.05% (0.66, 1.45) |
