# Supplementary material for: Analysis of global, regional, and national burden and attributable risk factors of acute lymphoblastic leukemia and acute myeloid leukemia from 1990 to 2021
Source: PLoS One. 2025 Sep 2;20(9):e0330479. doi: 10.1371/journal.pone.0330479 (PMC12404455; doi:10.1371/journal.pone.0330479)
Supplement: S6 Table — (DOCX) [file pone.0330479.s012.docx]

**Supplementary Table 6 EAPC of ASMR for acute leukemia in 204 countries and territories from 1990 to 2021**

| **Location** | EAPC (95%*CI*) | |
| --- | --- | --- |
|  | Acute lymphoblastic leukemia | Acute myeloid leukemia |
| Afghanistan | -0.40% (-0.51, -0.30) | 0.30% (0.24, 0.36) |
| Albania | -1.88% (-2.17, -1.59) | -0.36% (-0.51, -0.22) |
| Algeria | -1.74% (-1.80, -1.69) | -0.39% (-0.46, -0.31) |
| American Samoa | 0.79% (0.26, 1.31) | -2.92% (-3.28, -2.57) |
| Andorra | -2.09% (-2.20, -1.98) | -0.88% (-1.06, -0.71) |
| Angola | -0.89% (-0.98, -0.79) | -0.19% (-0.25, -0.13) |
| Antigua and Barbuda | -0.60% (-0.99, -0.21) | 1.13% (0.90, 1.37) |
| Argentina | -0.37% (-0.57, -0.17) | 0.25% (-0.02, 0.51) |
| Armenia | -3.53% (-3.92, -3.13) | 1.96% (1.24, 2.67) |
| Australia | -2.79% (-2.95, -2.62) | 0.77% (0.39, 1.14) |
| Austria | -2.31% (-2.43, -2.19) | 0.97% (0.73, 1.21) |
| Azerbaijan | -1.93% (-2.20, -1.67) | -0.57% (-0.71, -0.43) |
| Bahamas | -0.66% (-0.93, -0.40) | 0.99% (0.71, 1.28) |
| Bahrain | -2.36% (-2.56, -2.15) | -1.75% (-1.96, -1.54) |
| Bangladesh | -1.56% (-1.67, -1.46) | -0.46% (-0.53, -0.38) |
| Barbados | -1.22% (-1.58, -0.86) | 1.38% (1.18, 1.57) |
| Belarus | -4.13% (-4.67, -3.58) | 1.28% (0.80, 1.76) |
| Belgium | -2.74% (-3.03, -2.45) | 0.81% (0.50, 1.12) |
| Belize | -0.78% (-1.15, -0.41) | 1.66% (1.26, 2.07) |
| Benin | 0.45% (0.30, 0.61) | 0.63% (0.53, 0.73) |
| Bermuda | -2.83% (-2.96, -2.70) | -1.00% (-1.19, -0.81) |
| Bhutan | -1.10% (-1.27, -0.94) | 0.02% (-0.06, 0.10) |
| Bolivia (Plurinational State of) | -1.05% (-1.10, -1.00) | 0.16% (0.12, 0.19) |
| Bosnia and Herzegovina | -1.47% (-1.62, -1.33) | 0.37% (0.25, 0.50) |
| Botswana | -0.48% (-0.65, -0.30) | -0.23% (-0.36, -0.11) |
| Brazil | -0.52% (-0.70, -0.34) | 0.26% (0.13, 0.39) |
| Brunei Darussalam | -1.43% (-1.63, -1.23) | -0.46% (-0.60, -0.31) |
| Bulgaria | -2.04% (-2.43, -1.66) | 2.29% (1.95, 2.63) |
| Burkina Faso | 0.81% (0.65, 0.97) | 0.97% (0.88, 1.07) |
| Burundi | -0.63% (-0.75, -0.50) | -0.42% (-0.50, -0.34) |
| Cabo Verde | -0.88% (-1.09, -0.67) | 0.30% (0.03, 0.57) |
| Cambodia | -1.26% (-1.33, -1.2) | -0.07% (-0.11, -0.03) |
| Cameroon | 0.65% (0.45, 0.85) | 0.63% (0.52, 0.73) |
| Canada | -1.93% (-2.04, -1.82) | 0.78% (0.58, 0.97) |
| Central African Republic | -0.28% (-0.33, -0.22) | -0.37% (-0.40, -0.33) |
| Chad | 1.47% (1.31, 1.64) | 1.45% (1.32, 1.58) |
| Chile | -0.80% (-0.97, -0.64) | 0.44% (0.25, 0.64) |
| China | -2.7% (-2.79, -2.61) | -2.04% (-2.26, -1.81) |
| Colombia | -0.13% (-0.38, 0.11) | 0.52% (0.34, 0.69) |
| Comoros | -0.42% (-0.71, -0.12) | -0.18% (-0.37, 0.01) |
| Congo | -0.59% (-0.74, -0.45) | -0.59% (-0.69, -0.49) |
| Cook Islands | -2.53% (-2.73, -2.34) | -0.92% (-1.01, -0.84) |
| Costa Rica | 0.69% (0.51, 0.86) | 0.94% (0.72, 1.16) |
| Côte d'Ivoire | -0.10% (-0.25, 0.05) | -0.30% (-0.40, -0.20) |
| Croatia | -2.69% (-2.96, -2.42) | 1.41% (1.25, 1.58) |
| Cuba | -1.33% (-1.55, -1.12) | 0.08% (-0.12, 0.28) |
| Cyprus | -2.04% (-2.19, -1.90) | -0.46% (-0.61, -0.31) |
| Czechia | -2.94% (-3.35, -2.54) | 0.49% (0.20, 0.78) |
| Democratic People's Republic of Korea | -0.38% (-0.53, -0.23) | -0.67% (-0.71, -0.63) |
| Democratic Republic of the Congo | -0.16% (-0.25, -0.06) | -0.26% (-0.39, -0.13) |
| Denmark | -2.32% (-2.5, -2.13) | -0.6% (-0.83, -0.37) |
| Djibouti | -0.05% (-0.32, 0.22) | 0.19% (0.05, 0.33) |
| Dominica | 0.65% (0.53, 0.78) | 0.17% (0.11, 0.23) |
| Dominican Republic | -0.96% (-1.10, -0.82) | 0.56% (0.42, 0.71) |
| Ecuador | 1.21% (0.90, 1.52) | 1.8% (1.34, 2.26) |
| Egypt | 0.91% (0.53, 1.29) | 2.04% (1.71, 2.36) |
| El Salvador | -0.34% (-0.50, -0.18) | 0.59% (0.49, 0.68) |
| Equatorial Guinea | -1.74% (-1.96, -1.52) | -0.19% (-0.28, -0.10) |
| Eritrea | 0.08% (-0.03, 0.19) | 0.35% (0.26, 0.44) |
| Estonia | -3.64% (-4.03, -3.25) | 0.7% (0.17, 1.23) |
| Eswatini | 0.81% (0.44, 1.18) | 0.72% (0.34, 1.10) |
| Ethiopia | -2.22% (-2.37, -2.07) | -0.92% (-1.05, -0.79) |
| Fiji | -1.19% (-1.53, -0.84) | -0.09% (-0.25, 0.08) |
| Finland | -1.74% (-1.88, -1.60) | -0.29% (-0.45, -0.14) |
| France | -2.11% (-2.21, -2.01) | 0.90% (0.71, 1.09) |
| Gabon | -0.21% (-0.34, -0.07) | -0.34% (-0.43, -0.24) |
| Gambia | -0.75% (-0.99, -0.51) | -0.12% (-0.30, 0.06) |
| Georgia | -2.67% (-3.28, -2.05) | 2.40% (1.63, 3.17) |
| Germany | -1.99% (-2.09, -1.89) | 0.93% (0.86, 1.00) |
| Ghana | -3.12% (-3.60, -2.63) | -2.19% (-2.64, -1.75) |
| Greece | -1.56% (-1.65, -1.47) | 1.13% (0.97, 1.28) |
| Greenland | -3.41% (-3.59, -3.24) | -1.25% (-1.32, -1.19) |
| Grenada | -0.73% (-0.98, -0.48) | 1.06% (0.91, 1.21) |
| Guam | -2.29% (-3.12, -1.45) | 0.21% (-0.28, 0.71) |
| Guatemala | 1.37% (1.13, 1.60) | 0.62% (0.39, 0.86) |
| Guinea | -0.64% (-0.77, -0.52) | -0.25% (-0.29, -0.21) |
| Guinea-Bissau | 0.14% (-0.09, 0.38) | 0.77% (0.65, 0.89) |
| Guyana | 1.44% (0.97, 1.91) | 1.88% (1.63, 2.12) |
| Haiti | -0.65% (-0.75, -0.54) | -0.05% (-0.11, 0.02) |
| Honduras | -1.10% (-1.21, -1.00) | 0.18% (0.12, 0.24) |
| Hungary | -2.21% (-2.43, -1.99) | 0.44% (0.29, 0.58) |
| Iceland | -1.25% (-1.43, -1.07) | 0.44% (0.37, 0.52) |
| India | -1.70% (-1.81, -1.59) | 0.14% (0.04, 0.23) |
| Indonesia | -0.59% (-0.71, -0.47) | 0.21% (0.10, 0.32) |
| Iran (Islamic Republic of) | -1.90% (-2.05, -1.74) | -0.55% (-0.66, -0.45) |
| Iraq | -1.54% (-1.66, -1.41) | -0.12% (-0.20, -0.04) |
| Ireland | -3.02% (-3.29, -2.76) | 0.03% (-0.27, 0.32) |
| Israel | -2.59% (-2.84, -2.35) | 0.25% (0.02, 0.47) |
| Italy | -2.28% (-2.44, -2.12) | 1.06% (0.85, 1.27) |
| Jamaica | -1.02% (-1.33, -0.72) | 2.35% (1.99, 2.72) |
| Japan | -2.36% (-2.52, -2.20) | -0.29% (-0.53, -0.06) |
| Jordan | -3.24% (-3.62, -2.85) | -1.35% (-1.57, -1.13) |
| Kazakhstan | -2.17% (-2.49, -1.85) | -0.66% (-0.99, -0.32) |
| Kenya | 0.52% (0.31, 0.73) | 1.05% (0.92, 1.18) |
| Kiribati | -0.12% (-0.26, 0.02) | 0.16% (0.09, 0.22) |
| Kuwait | -3.30% (-3.71, -2.89) | -0.56% (-1.13, 0.01) |
| Kyrgyzstan | -2.56% (-2.91, -2.22) | 1.58% (1.26, 1.91) |
| Lao People's Democratic Republic | -1.57% (-1.66, -1.48) | -0.38% (-0.44, -0.32) |
| Latvia | -3.12% (-3.33, -2.92) | -0.72% (-1.08, -0.36) |
| Lebanon | -1.57% (-1.61, -1.52) | -0.20% (-0.32, -0.08) |
| Lesotho | 2.31% (1.96, 2.66) | 1.96% (1.70, 2.23) |
| Liberia | 0.23% (-0.13, 0.60) | 0.85% (0.64, 1.06) |
| Libya | 0.92% (0.63, 1.20) | 0.05% (-0.07, 0.18) |
| Lithuania | -2.35% (-2.54, -2.15) | 2.16% (1.78, 2.55) |
| Luxembourg | -4.01% (-4.13, -3.89) | 0.30% (0.12, 0.47) |
| Madagascar | -0.25% (-0.34, -0.16) | -0.29% (-0.40, -0.18) |
| Malawi | -1.30% (-1.40, -1.20) | -0.65% (-0.73, -0.56) |
| Malaysia | -1.19% (-1.33, -1.05) | -0.11% (-0.26, 0.04) |
| Maldives | -3.00% (-3.09, -2.91) | -1.74% (-1.81, -1.67) |
| Mali | -1.04% (-1.14, -0.93) | -0.52% (-0.58, -0.46) |
| Malta | -1.88% (-2.08, -1.67) | 0.57% (0.34, 0.80) |
| Marshall Islands | -0.05% (-0.31, 0.22) | 0.36% (0.29, 0.43) |
| Mauritania | -0.01% (-0.18, 0.17) | 0.47% (0.38, 0.56) |
| Mauritius | 3.04% (-1.39, 7.67) | 5.15% (1.91, 8.50) |
| Mexico | 0.22% (0.07, 0.38) | 0.12% (0.02, 0.23) |
| Micronesia (Federated States of) | -0.65% (-0.73, -0.58) | -0.22% (-0.24, -0.19) |
| Monaco | -0.92% (-1.10, -0.73) | 0.98% (0.77, 1.19) |
| Mongolia | -1.93% (-2.15, -1.72) | -0.10% (-0.22, 0.01) |
| Montenegro | -1.72% (-2.01, -1.43) | 0.14% (0.03, 0.26) |
| Morocco | -1.14% (-1.26, -1.02) | 0.17% (0.12, 0.22) |
| Mozambique | -0.68% (-0.84, -0.52) | 0.06% (-0.02, 0.15) |
| Myanmar | -1.93% (-2.09, -1.77) | -0.76% (-0.86, -0.65) |
| Namibia | -0.12% (-0.25, 0.01) | -0.01% (-0.19, 0.16) |
| Nauru | 0.20% (-0.13, 0.53) | -0.43% (-0.49, -0.37) |
| Nepal | -1.21% (-1.33, -1.09) | 0.28% (0.12, 0.43) |
| Netherlands | -2.37% (-2.66, -2.07) | 0.26% (0.10, 0.43) |
| New Zealand | -2.74% (-3.09, -2.39) | -0.73% (-1.08, -0.38) |
| Nicaragua | -0.81% (-1.00, -0.61) | 0.18% (0.02, 0.35) |
| Niger | -0.31% (-0.46, -0.16) | 0.51% (0.43, 0.59) |
| Nigeria | -0.28% (-0.37, -0.19) | 0.06% (-0.01, 0.14) |
| Niue | 0.54% (-0.17, 1.24) | 0.26% (-0.13, 0.65) |
| North Macedonia | -1.83% (-2.03, -1.63) | -0.35% (-0.56, -0.14) |
| Northern Mariana Islands | -1.63% (-1.89, -1.37) | -3.25% (-3.55, -2.95) |
| Norway | -1.67% (-1.96, -1.38) | -0.03% (-0.18, 0.11) |
| Oman | -2.05% (-2.21, -1.89) | -0.65% (-0.82, -0.47) |
| Pakistan | -0.26% (-0.38, -0.14) | 0.27% (0.15, 0.40) |
| Palau | -0.33% (-0.46, -0.19) | -0.10% (-0.16, -0.03) |
| Palestine | -1.7% (-1.85, -1.56) | -0.75% (-0.84, -0.65) |
| Panama | 0.43% (0.26, 0.60) | 1.04% (0.86, 1.23) |
| Papua New Guinea | -0.32% (-0.51, -0.13) | -0.04% (-0.08, 0) |
| Paraguay | 0.09% (-0.15, 0.33) | 1.09% (0.91, 1.28) |
| Peru | -0.32% (-0.45, -0.19) | 0.28% (0.15, 0.42) |
| Philippines | -0.73% (-0.82, -0.63) | -0.30% (-0.37, -0.22) |
| Poland | -2.51% (-2.74, -2.28) | 0.54% (0.24, 0.84) |
| Portugal | -3.33% (-3.58, -3.08) | 0.44% (0.26, 0.62) |
| Puerto Rico | -1.96% (-2.24, -1.69) | -0.15% (-0.34, 0.04) |
| Qatar | -2.66% (-2.99, -2.32) | -2.06% (-2.40, -1.71) |
| Republic of Korea | -3.85% (-4.03, -3.67) | -0.83% (-1.00, -0.66) |
| Republic of Moldova | -2.99% (-3.13, -2.84) | -0.41% (-0.88, 0.06) |
| Romania | -1.99% (-2.19, -1.80) | 1.46% (1.32, 1.60) |
| Russian Federation | -2.67% (-2.97, -2.38) | 0.12% (-0.08, 0.33) |
| Rwanda | -1.79% (-1.95, -1.62) | -1.06% (-1.21, -0.91) |
| Saint Kitts and Nevis | -1.13% (-1.36, -0.89) | 1.11% (0.89, 1.34) |
| Saint Lucia | -1.09% (-1.32, -0.86) | -0.04% (-0.22, 0.14) |
| Saint Vincent and the Grenadines | -0.46% (-0.84, -0.07) | 0.76% (0.58, 0.93) |
| Samoa | -0.12% (-0.16, -0.08) | -0.25% (-0.34, -0.17) |
| San Marino | -1.22% (-1.47, -0.98) | -0.85% (-1.18, -0.52) |
| Sao Tome and Principe | -1.77% (-1.96, -1.58) | -0.31% (-0.43, -0.19) |
| Saudi Arabia | -0.50% (-0.80, -0.19) | 1.19% (0.76, 1.61) |
| Senegal | 0.14% (-0.10, 0.37) | 0.93% (0.78, 1.09) |
| Serbia | -2.75% (-2.94, -2.56) | -0.08% (-0.17, 0) |
| Seychelles | -0.72% (-1.03, -0.41) | -0.43% (-0.55, -0.32) |
| Sierra Leone | 0.59% (0.40, 0.78) | 1.07% (0.92, 1.22) |
| Singapore | -2.57% (-2.92, -2.22) | -0.82% (-1.09, -0.54) |
| Slovakia | -2.15% (-2.24, -2.06) | -0.31% (-0.39, -0.22) |
| Slovenia | -2.38% (-2.65, -2.12) | 0.86% (0.61, 1.12) |
| Solomon Islands | 0.06% (-0.05, 0.18) | 0.21% (0.15, 0.27) |
| Somalia | 0.15% (-0.03, 0.34) | -0.04% (-0.11, 0.03) |
| South Africa | -0.10% (-0.33, 0.13) | 0.16% (0.01, 0.30) |
| South Sudan | 0.75% (0.40, 1.10) | 0.16% (0.02, 0.30) |
| Spain | -3.02% (-3.10, -2.94) | 0.72% (0.58, 0.86) |
| Sri Lanka | -2.32% (-2.55, -2.09) | -0.93% (-1.10, -0.76) |
| Sudan | -1.01% (-1.08, -0.93) | 0.10% (0.04, 0.16) |
| Suriname | -0.57% (-0.78, -0.36) | 0.47% (0.33, 0.61) |
| Sweden | -2.40% (-2.61, -2.19) | -0.12% (-0.27, 0.03) |
| Switzerland | -3.03% (-3.23, -2.83) | 0.15% (-0.15, 0.46) |
| Syrian Arab Republic | -2.12% (-2.42, -1.81) | -0.9% (-1.05, -0.74) |
| Taiwan (Province of China) | -0.13% (-0.38, 0.11) | 1.07% (0.77, 1.36) |
| Tajikistan | -1.90% (-2.09, -1.71) | -0.92% (-1.05, -0.79) |
| Thailand | -1.92% (-2.13, -1.70) | 0.56% (0.46, 0.65) |
| Timor-Leste | -1.04% (-1.29, -0.78) | 0% (-0.20, 0.20) |
| Togo | 0.51% (0.37, 0.65) | 0.99% (0.90, 1.08) |
| Tokelau | -0.05% (-0.99, 0.90) | 0.23% (-0.35, 0.82) |
| Tonga | -0.07% (-0.29, 0.14) | 0.35% (0.24, 0.46) |
| Trinidad and Tobago | -0.88% (-1.07, -0.68) | 0.68% (0.55, 0.80) |
| Tunisia | -1.77% (-1.82, -1.72) | -0.54% (-0.58, -0.51) |
| Türkiye | -2.72% (-2.82, -2.62) | -1.37% (-1.59, -1.15) |
| Turkmenistan | -1.46% (-1.75, -1.17) | -0.01% (-0.29, 0.27) |
| Tuvalu | -1.15% (-1.21, -1.10) | -0.11% (-0.13, -0.09) |
| Uganda | -0.41% (-0.57, -0.24) | 0.36% (0.24, 0.47) |
| Ukraine | -3.17% (-3.46, -2.87) | -2.24% (-2.49, -1.99) |
| United Arab Emirates | -1.64% (-1.94, -1.35) | 0.16% (-0.20, 0.51) |
| United Kingdom | -2.37% (-2.56, -2.18) | 0.44% (0.31, 0.58) |
| United Republic of Tanzania | -0.22% (-0.36, -0.07) | -0.07% (-0.11, -0.02) |
| United States of America | -1.54% (-1.63, -1.46) | 0.08% (-0.12, 0.28) |
| United States Virgin Islands | -1.35% (-1.56, -1.14) | -0.72% (-0.89, -0.54) |
| Uruguay | -0.55% (-0.73, -0.37) | 0.28% (0.15, 0.41) |
| Uzbekistan | -1.89% (-2.13, -1.65) | -0.59% (-0.90, -0.29) |
| Vanuatu | 0.06% (-0.13, 0.26) | 0.02% (-0.04, 0.08) |
| Venezuela (Bolivarian Republic of) | 0.21% (-0.04, 0.46) | 0.60% (0.42, 0.78) |
| Viet Nam | -1.24% (-1.30, -1.19) | -0.49% (-0.59, -0.40) |
| Yemen | -0.86% (-0.96, -0.75) | 0.16% (0.09, 0.24) |
| Zambia | -1.56% (-1.80, -1.32) | 0.54% (0.43, 0.64) |
| Zimbabwe | 2.18% (1.72, 2.66) | 1.00% (0.62, 1.38) |
