# Supplementary material for: Analysis of global, regional, and national burden and attributable risk factors of acute lymphoblastic leukemia and acute myeloid leukemia from 1990 to 2021
Source: PLoS One. 2025 Sep 2;20(9):e0330479. doi: 10.1371/journal.pone.0330479 (PMC12404455; doi:10.1371/journal.pone.0330479)
Supplement: S7 Table — (DOCX) [file pone.0330479.s013.docx]

**Supplementary Table 7 EAPC of ASDR for acute leukemia in 204 countries and territories from 1990 to 2021**

| Location | EAPC (95%*CI*) | |
| --- | --- | --- |
|  | Acute lymphoblastic leukemia | Acute myeloid leukemia |
| Afghanistan | -0.54% (-0.65, -0.42) | 0.27% (0.20, 0.34) |
| Albania | -2.14% (-2.51, -1.77) | -0.78% (-0.98, -0.57) |
| Algeria | -2.16% (-2.21, -2.1) | -0.98% (-1.03, -0.92) |
| American Samoa | 0.98% (0.20, 1.77) | -2.22% (-2.49, -1.94) |
| Andorra | -2.24% (-2.35, -2.13) | -1.12% (-1.26, -0.97) |
| Angola | -1.09% (-1.22, -0.96) | -0.26% (-0.34, -0.17) |
| Antigua and Barbuda | -0.82% (-1.20, -0.43) | 0.80% (0.53, 1.07) |
| Argentina | -0.70% (-0.89, -0.51) | -0.31% (-0.55, -0.07) |
| Armenia | -3.8% (-4.18, -3.42) | 1.32% (0.55, 2.09) |
| Australia | -3.02% (-3.17, -2.87) | 0.09% (-0.22, 0.41) |
| Austria | -2.64% (-2.78, -2.51) | 0.18% (-0.05, 0.42) |
| Azerbaijan | -2.10% (-2.40, -1.80) | -0.89% (-1.03, -0.74) |
| Bahamas | -0.94% (-1.21, -0.66) | 0.68% (0.38, 0.98) |
| Bahrain | -2.38% (-2.57, -2.20) | -1.92% (-2.09, -1.76) |
| Bangladesh | -1.75% (-1.86, -1.63) | -0.72% (-0.79, -0.66) |
| Barbados | -1.65% (-2.04, -1.26) | 0.84% (0.64, 1.05) |
| Belarus | -4.81% (-5.35, -4.26) | 0.53% (-0.06, 1.12) |
| Belgium | -2.99% (-3.25, -2.73) | 0.16% (-0.08, 0.41) |
| Belize | -1.41% (-1.74, -1.07) | 1.05% (0.72, 1.38) |
| Benin | 0.41% (0.24, 0.57) | 0.53% (0.42, 0.63) |
| Bermuda | -2.09% (-2.25, -1.93) | -1.13% (-1.29, -0.96) |
| Bhutan | -1.32% (-1.55, -1.09) | -0.36% (-0.50, -0.21) |
| Bolivia (Plurinational State of) | -1.43% (-1.49, -1.36) | -0.27% (-0.3, -0.24) |
| Bosnia and Herzegovina | -1.81% (-1.99, -1.64) | -0.04% (-0.18, 0.10) |
| Botswana | -0.2% (-0.38, -0.02) | -0.08% (-0.21, 0.06) |
| Brazil | -0.71% (-0.93, -0.50) | -0.35% (-0.49, -0.20) |
| Brunei Darussalam | -1.63% (-1.83, -1.44) | -0.79% (-0.93, -0.65) |
| Bulgaria | -2.35% (-2.72, -1.98) | 1.69% (1.34, 2.04) |
| Burkina Faso | 0.80% (0.62, 0.97) | 1.02% (0.88, 1.16) |
| Burundi | -0.76% (-0.94, -0.57) | -0.55% (-0.67, -0.42) |
| Cabo Verde | -1.06% (-1.25, -0.86) | -0.23% (-0.46, 0.01) |
| Cambodia | -1.54% (-1.63, -1.45) | -0.29% (-0.34, -0.25) |
| Cameroon | 0.65% (0.43, 0.87) | 0.64% (0.52, 0.76) |
| Canada | -2.19% (-2.31, -2.07) | 0.12% (-0.02, 0.27) |
| Central African Republic | -0.36% (-0.42, -0.29) | -0.36% (-0.41, -0.31) |
| Chad | 1.42% (1.25, 1.59) | 1.37% (1.25, 1.49) |
| Chile | -1.27% (-1.44, -1.09) | -0.31% (-0.47, -0.15) |
| China | -3.31% (-3.44, -3.18) | -2.81% (-3.07, -2.55) |
| Colombia | -0.25% (-0.56, 0.06) | 0.07% (-0.13, 0.27) |
| Comoros | -0.54% (-0.87, -0.21) | -0.31% (-0.58, -0.04) |
| Congo | -0.71% (-0.88, -0.53) | -0.60% (-0.71, -0.49) |
| Cook Islands | -3.52% (-3.94, -3.10) | -1.22% (-1.37, -1.07) |
| Costa Rica | 0.52% (0.33, 0.71) | 0.80% (0.55, 1.05) |
| Côte d'Ivoire | -0.07% (-0.24, 0.11) | -0.25% (-0.38, -0.12) |
| Croatia | -2.59% (-2.78, -2.40) | 1.06% (0.86, 1.26) |
| Cuba | -1.75% (-1.96, -1.54) | -0.41% (-0.64, -0.19) |
| Cyprus | -1.76% (-2.04, -1.48) | -0.64% (-0.77, -0.50) |
| Czechia | -3.03% (-3.34, -2.72) | 0% (-0.27, 0.26) |
| Democratic People's Republic of Korea | -0.60% (-0.78, -0.43) | -1.10% (-1.15, -1.06) |
| Democratic Republic of the Congo | -0.36% (-0.48, -0.24) | -0.32% (-0.46, -0.18) |
| Denmark | -2.53% (-2.80, -2.27) | -1.37% (-1.59, -1.14) |
| Djibouti | -0.28% (-0.59, 0.03) | -0.07% (-0.27, 0.13) |
| Dominica | 0.96% (0.80, 1.13) | 0.55% (0.45, 0.64) |
| Dominican Republic | -1.37% (-1.54, -1.19) | 0.06% (-0.10, 0.22) |
| Ecuador | 0.84% (0.47, 1.21) | 1.33% (0.83, 1.84) |
| Egypt | 0.26% (-0.09, 0.61) | 1.49% (1.21, 1.77) |
| El Salvador | -0.76% (-0.94, -0.58) | 0.16% (0.05, 0.27) |
| Equatorial Guinea | -2.00% (-2.21, -1.79) | -0.32% (-0.41, -0.23) |
| Eritrea | -0.04% (-0.17, 0.09) | 0.29% (0.17, 0.40) |
| Estonia | -4.32% (-4.59, -4.04) | -0.38% (-0.76, -0.01) |
| Eswatini | 0.86% (0.53, 1.18) | 0.88% (0.50, 1.27) |
| Ethiopia | -2.18% (-2.34, -2.03) | -1.14% (-1.31, -0.97) |
| Fiji | -1.59% (-1.91, -1.27) | -0.03% (-0.22, 0.16) |
| Finland | -1.8% (-1.96, -1.64) | -0.58% (-0.70, -0.47) |
| France | -2.50% (-2.60, -2.40) | 0.16% (0, 0.32) |
| Gabon | -0.21% (-0.36, -0.06) | -0.30% (-0.40, -0.19) |
| Gambia | -0.94% (-1.20, -0.68) | -0.38% (-0.61, -0.14) |
| Georgia | -3.65% (-4.25, -3.05) | 1.10% (0.38, 1.82) |
| Germany | -2.29% (-2.48, -2.09) | 0.10% (0.02, 0.17) |
| Ghana | -3.18% (-3.67, -2.69) | -2.35% (-2.80, -1.89) |
| Greece | -1.69% (-1.81, -1.56) | 0.89% (0.78, 1.01) |
| Greenland | -3.51% (-3.69, -3.33) | -1.52% (-1.61, -1.43) |
| Grenada | -1.05% (-1.30, -0.80) | 0.74% (0.59, 0.90) |
| Guam | -1.79% (-2.70, -0.87) | 0.44% (-0.04, 0.92) |
| Guatemala | 1.02% (0.80, 1.24) | 0.14% (-0.07, 0.36) |
| Guinea | -0.79% (-0.93, -0.65) | -0.48% (-0.53, -0.42) |
| Guinea-Bissau | -0.07% (-0.34, 0.19) | 0.46% (0.31, 0.61) |
| Guyana | 1.22% (0.74, 1.70) | 1.71% (1.43, 1.98) |
| Haiti | -0.77% (-0.90, -0.65) | -0.17% (-0.26, -0.08) |
| Honduras | -1.98% (-2.11, -1.86) | -0.70% (-0.76, -0.63) |
| Hungary | -2.63% (-2.84, -2.43) | 0.08% (-0.06, 0.23) |
| Iceland | -1.49% (-1.73, -1.25) | -0.02% (-0.08, 0.04) |
| India | -2.12% (-2.28, -1.95) | -0.25% (-0.35, -0.15) |
| Indonesia | -0.86% (-0.97, -0.74) | -0.06% (-0.19, 0.06) |
| Iran (Islamic Republic of) | -2.24% (-2.47, -2.02) | -0.90% (-1.02, -0.79) |
| Iraq | -1.77% (-1.95, -1.59) | -0.34% (-0.39, -0.28) |
| Ireland | -3.15% (-3.45, -2.85) | -0.36% (-0.68, -0.04) |
| Israel | -2.96% (-3.19, -2.73) | -0.34% (-0.56, -0.11) |
| Italy | -2.78% (-2.96, -2.60) | 0.11% (-0.10, 0.31) |
| Jamaica | -1.77% (-2.08, -1.46) | 1.43% (1.13, 1.73) |
| Japan | -2.78% (-2.94, -2.62) | -1.1% (-1.31, -0.90) |
| Jordan | -3.24% (-3.60, -2.88) | -1.58% (-1.79, -1.38) |
| Kazakhstan | -2.57% (-2.92, -2.22) | -1.31% (-1.69, -0.93) |
| Kenya | 0.38% (0.13, 0.64) | 0.99% (0.83, 1.15) |
| Kiribati | -0.58% (-0.74, -0.41) | 0.04% (-0.05, 0.12) |
| Kuwait | -3.41% (-3.79, -3.02) | -0.99% (-1.55, -0.42) |
| Kyrgyzstan | -2.99% (-3.33, -2.65) | 0.90% (0.57, 1.23) |
| Lao People's Democratic Republic | -1.68% (-1.78, -1.58) | -0.46% (-0.52, -0.39) |
| Latvia | -3.58% (-3.79, -3.37) | -1.41% (-1.81, -1.02) |
| Lebanon | -1.79% (-1.83, -1.76) | -0.52% (-0.62, -0.43) |
| Lesotho | 2.41% (2.07, 2.76) | 2.22% (1.93, 2.52) |
| Liberia | -0.01% (-0.44, 0.42) | 0.65% (0.32, 0.99) |
| Libya | 0.60% (0.32, 0.88) | -0.08% (-0.19, 0.04) |
| Lithuania | -3.08% (-3.36, -2.81) | 1.31% (0.96, 1.66) |
| Luxembourg | -4.64% (-4.84, -4.45) | -0.68% (-0.84, -0.53) |
| Madagascar | -0.39% (-0.50, -0.27) | -0.35% (-0.47, -0.23) |
| Malawi | -1.63% (-1.75, -1.52) | -1.00% (-1.09, -0.91) |
| Malaysia | -1.54% (-1.78, -1.30) | -0.43% (-0.54, -0.32) |
| Maldives | -2.96% (-3.06, -2.86) | -1.79% (-1.86, -1.72) |
| Mali | -1.17% (-1.30, -1.04) | -0.80% (-0.88, -0.72) |
| Malta | -1.78% (-2.03, -1.52) | 0.60% (0.40, 0.80) |
| Marshall Islands | 0.19% (-0.15, 0.52) | 0.29% (0.19, 0.39) |
| Mauritania | -0.14% (-0.35, 0.07) | 0.27% (0.15, 0.39) |
| Mauritius | 2.10% (-1.75, 6.11) | 3.49% (0.82, 6.22) |
| Mexico | -0.19% (-0.35, -0.03) | -0.38% (-0.48, -0.28) |
| Micronesia (Federated States of) | -1.12% (-1.22, -1.01) | -0.32% (-0.35, -0.28) |
| Monaco | -1.31% (-1.53, -1.08) | 0.66% (0.48, 0.84) |
| Mongolia | -2.19% (-2.46, -1.92) | -0.32% (-0.47, -0.16) |
| Montenegro | -2.22% (-2.58, -1.86) | -0.31% (-0.48, -0.13) |
| Morocco | -1.57% (-1.69, -1.45) | -0.24% (-0.30, -0.19) |
| Mozambique | -1.06% (-1.23, -0.88) | -0.16% (-0.26, -0.07) |
| Myanmar | -2.06% (-2.23, -1.89) | -0.99% (-1.11, -0.86) |
| Namibia | 0.02% (-0.07, 0.11) | 0.07% (-0.08, 0.22) |
| Nauru | -0.14% (-0.56, 0.29) | -0.27% (-0.37, -0.17) |
| Nepal | -1.58% (-1.69, -1.48) | -0.15% (-0.29, 0) |
| Netherlands | -3.02% (-3.32, -2.72) | -0.45% (-0.62, -0.27) |
| New Zealand | -2.78% (-3.06, -2.49) | -0.93% (-1.25, -0.62) |
| Nicaragua | -1.38% (-1.58, -1.17) | -0.44% (-0.58, -0.30) |
| Niger | -0.60% (-0.77, -0.43) | -0.06% (-0.18, 0.06) |
| Nigeria | -0.25% (-0.35, -0.16) | 0.03% (-0.07, 0.14) |
| Niue | 0.85% (-0.12, 1.83) | 0.52% (-0.10, 1.15) |
| North Macedonia | -2.35% (-2.57, -2.12) | -0.81% (-0.98, -0.65) |
| Northern Mariana Islands | -1.74% (-2.00, -1.47) | -2.68% (-2.87, -2.49) |
| Norway | -1.88% (-2.15, -1.61) | -0.74% (-0.87, -0.60) |
| Oman | -2.29% (-2.51, -2.07) | -0.87% (-1.10, -0.65) |
| Pakistan | -0.19% (-0.31, -0.07) | 0.37% (0.25, 0.49) |
| Palau | -0.06% (-0.20, 0.07) | -0.15% (-0.21, -0.10) |
| Palestine | -1.82% (-2.00, -1.64) | -0.91% (-1.00, -0.82) |
| Panama | 0.06% (-0.11, 0.24) | 0.74% (0.57, 0.91) |
| Papua New Guinea | -0.27% (-0.51, -0.04) | -0.10% (-0.16, -0.05) |
| Paraguay | -0.33% (-0.61, -0.06) | 0.64% (0.45, 0.84) |
| Peru | -0.60% (-0.73, -0.48) | -0.15% (-0.26, -0.03) |
| Philippines | -0.74% (-0.88, -0.59) | -0.27% (-0.33, -0.21) |
| Poland | -3.01% (-3.24, -2.78) | -0.31% (-0.62, -0.01) |
| Portugal | -3.83% (-4.10, -3.56) | -0.46% (-0.64, -0.28) |
| Puerto Rico | -2.51% (-2.77, -2.25) | -0.56% (-0.76, -0.36) |
| Qatar | -2.57% (-2.84, -2.30) | -2.12% (-2.42, -1.83) |
| Republic of Korea | -4.15% (-4.46, -3.84) | -1.83% (-1.98, -1.69) |
| Republic of Moldova | -3.57% (-3.74, -3.39) | -1.03% (-1.48, -0.58) |
| Romania | -2.91% (-3.15, -2.67) | 0.32% (0.18, 0.46) |
| Russian Federation | -3.38% (-3.67, -3.09) | -0.86% (-1.02, -0.70) |
| Rwanda | -1.93% (-2.11, -1.75) | -1.21% (-1.33, -1.08) |
| Saint Kitts and Nevis | -1.28% (-1.50, -1.05) | 0.76% (0.55, 0.98) |
| Saint Lucia | -1.17% (-1.43, -0.90) | 0.03% (-0.19, 0.24) |
| Saint Vincent and the Grenadines | -0.72% (-1.09, -0.36) | 0.45% (0.29, 0.61) |
| Samoa | -0.49% (-0.55, -0.44) | -0.41% (-0.52, -0.30) |
| San Marino | -1.50% (-1.69, -1.31) | -1.01% (-1.29, -0.72) |
| Sao Tome and Principe | -2.16% (-2.36, -1.96) | -0.75% (-0.89, -0.62) |
| Saudi Arabia | -1.08% (-1.33, -0.82) | 0.86% (0.51, 1.21) |
| Senegal | -0.11% (-0.37, 0.15) | 0.64% (0.44, 0.83) |
| Serbia | -3.66% (-3.91, -3.41) | -0.73% (-0.84, -0.62) |
| Seychelles | -0.70% (-1.14, -0.26) | -0.56% (-0.70, -0.42) |
| Sierra Leone | 0.39% (0.19, 0.59) | 0.91% (0.73, 1.09) |
| Singapore | -2.61% (-3.02, -2.21) | -1.14% (-1.35, -0.92) |
| Slovakia | -2.14% (-2.23, -2.05) | -0.55% (-0.64, -0.46) |
| Slovenia | -3.03% (-3.26, -2.81) | 0.16% (-0.08, 0.40) |
| Solomon Islands | -0.14% (-0.31, 0.02) | 0.26% (0.17, 0.36) |
| Somalia | -0.04% (-0.28, 0.20) | -0.12% (-0.24, -0.01) |
| South Africa | -0.43% (-0.66, -0.20) | -0.16% (-0.30, -0.03) |
| South Sudan | 0.77% (0.35, 1.18) | 0.20% (-0.03, 0.43) |
| Spain | -3.27% (-3.37, -3.17) | -0.19% (-0.30, -0.08) |
| Sri Lanka | -2.71% (-3.00, -2.42) | -1.55% (-1.72, -1.39) |
| Sudan | -1.18% (-1.28, -1.08) | 0.02% (-0.04, 0.09) |
| Suriname | -0.75% (-1.00, -0.51) | 0.24% (0.09, 0.39) |
| Sweden | -2.78% (-3.06, -2.49) | -0.61% (-0.75, -0.47) |
| Switzerland | -3.44% (-3.66, -3.22) | -0.63% (-0.92, -0.34) |
| Syrian Arab Republic | -2.50% (-2.85, -2.15) | -1.31% (-1.49, -1.13) |
| Taiwan (Province of China) | -0.38% (-0.66, -0.10) | 0.60% (0.43, 0.77) |
| Tajikistan | -2.04% (-2.29, -1.79) | -1.09% (-1.28, -0.89) |
| Thailand | -1.77% (-1.97, -1.57) | 0.28% (0.12, 0.43) |
| Timor-Leste | -1.39% (-1.70, -1.09) | -0.20% (-0.50, 0.09) |
| Togo | 0.34% (0.19, 0.48) | 0.75% (0.66, 0.84) |
| Tokelau | -0.03% (-1.34, 1.29) | 0.52% (-0.43, 1.48) |
| Tonga | -0.42% (-0.71, -0.13) | 0.35% (0.20, 0.50) |
| Trinidad and Tobago | -1.12% (-1.30, -0.94) | 0.45% (0.32, 0.59) |
| Tunisia | -2.14% (-2.22, -2.07) | -0.78% (-0.81, -0.74) |
| Türkiye | -3.14% (-3.19, -3.08) | -2.03% (-2.20, -1.87) |
| Turkmenistan | -1.58% (-1.91, -1.26) | -0.24% (-0.56, 0.09) |
| Tuvalu | -1.59% (-1.66, -1.52) | -0.34% (-0.38, -0.31) |
| Uganda | -0.41% (-0.57, -0.25) | 0.46% (0.34, 0.58) |
| Ukraine | -3.76% (-4.11, -3.42) | -3.01% (-3.32, -2.71) |
| United Arab Emirates | -2.06% (-2.34, -1.77) | -0.53% (-0.79, -0.27) |
| United Kingdom | -2.60% (-2.79, -2.40) | -0.12% (-0.27, 0.04) |
| United Republic of Tanzania | -0.26% (-0.44, -0.08) | 0.02% (-0.07, 0.12) |
| United States of America | -1.88% (-1.97, -1.78) | -0.49% (-0.67, -0.32) |
| United States Virgin Islands | -1.42% (-1.67, -1.17) | -0.60% (-0.78, -0.42) |
| Uruguay | -1.15% (-1.35, -0.94) | -0.24% (-0.40, -0.08) |
| Uzbekistan | -1.84% (-2.07, -1.61) | -0.78% (-1.09, -0.48) |
| Vanuatu | -0.14% (-0.43, 0.15) | 0.08% (-0.02, 0.17) |
| Venezuela (Bolivarian Republic of) | -0.04% (-0.34, 0.27) | 0.19% (0.07, 0.31) |
| Viet Nam | -1.11% (-1.17, -1.05) | -0.24% (-0.27, -0.20) |
| Yemen | -1.07% (-1.20, -0.94) | 0.07% (-0.04, 0.17) |
| Zambia | -1.49% (-1.70, -1.28) | 0.52% (0.38, 0.66) |
| Zimbabwe | 2.63% (2.11, 3.14) | 1.46% (1.02, 1.90) |
