# Supplementary material for: Analysis of global, regional, and national burden and attributable risk factors of acute lymphoblastic leukemia and acute myeloid leukemia from 1990 to 2021
Source: PLoS One. 2025 Sep 2;20(9):e0330479. doi: 10.1371/journal.pone.0330479 (PMC12404455; doi:10.1371/journal.pone.0330479)
Supplement: S8 Table — (DOCX) [file pone.0330479.s014.docx]

**Supplementary Table 8 Percentage of acute leukemia deaths and DALYs attributable to risk factors in 1990 and 2021**

| Cancer |  | 1990 |  |  | 2021 |  |
| --- | --- | --- | --- | --- | --- | --- |
|  | Leading risk | Percentage of death  (95% UI) | Percentage of DALYs  (95% UI) | Leading risk | Percentage of death  (95% UI) | Percentage of DALYs  (95% UI) |
| Acute lymphoblastic leukemia | Smoking | 3.29(1.22～5.58) | 1.39(0.52～2.36) | Smoking | 5.68(2.07～9.35) | 2.70(0.99～4.49) |
|  | High BMI | 2.60(1.83～3.52) | 1.60(1.10～2.17) | High BMI | 5.78(4.32～7.38) | 4.02(2.99～5.17) |
|  | Occupational exposure to benzene | 0.43(0.12～0.71) | 0.36(0.10～0.60) | Occupational exposure to benzene | 0.74(0.22～1.21) | 0.73(0.21～1.19) |
|  | Occupational exposure to formaldehyde | 0.16(0.13～0.20) | 0.14(0.11～0.18) | Occupational exposure to formaldehyde | 0.28(0.23～0.33) | 0.27(0.22～0.33) |
| Acute myeloid leukemia | Smoking | 10.87(3.93～17.85) | 5.90(2.13～9.78) | Smoking | 11.47(4.05～19.61) | 7.47(2.76～12.54) |
|  | High BMI | 5.95(4.21～7.78) | 4.20(2.77～5.72) | High BMI | 9.18(6.79～11.50) | 7.76(5.79～9.72) |
|  | Occupational exposure to benzene | 0.52(0.17～0.87) | 0.58(0.18～1.00) | Occupational exposure to benzene | 0.59(0.18～0.96) | 0.83(0.24～1.36) |
|  | Occupational exposure to formaldehyde | 0.16(0.13～0.19) | 0.18(0.15～0.23) | Occupational exposure to formaldehyde | 0.18(0.15～0.22) | 0.26(0.22～0.32) |
